# Machine Learning-Driven Personalized Risk Prediction: Developing an Explainable Sarcopenia Model for Older European Adults with Arthritis

**DOI:** 10.3390/jcm15031022

**Published:** 2026-01-27

**Authors:** Xiao Xu

**Affiliations:** 1 Research Center of Molecular Medicine (PhD Lab), Faculty of Nursing, Nantong Health College of Jiangsu Province, Nantong 226001, China; xuxiao1@nthvc.cn; 2 Uptown Campus, University at Albany, Albany, NY 12222, USA

**Keywords:** machine learning, sarcopenia, arthritis

## Abstract

**Objectives**: This study aimed to develop and validate an explainable machine learning (ML) model to predict the risk of sarcopenia in older European adults with arthritis, providing a practical tool for early and precise screening in clinical settings. **Methods**: We analyzed data from the English Longitudinal Study of Aging (ELSA) and the Survey of Health, Aging and Retirement in Europe (SHARE). The final analysis included 1959 participants aged ≥65 years. The ELSA dataset was divided into a training set (n = 1371) and an internal validation set (n = 588), while the SHARE dataset (n = 1001) served as an independent external test cohort. From an initial pool of 33 variables, nine core predictors were identified using ensemble feature selection techniques. Six ML algorithms were compared, with model performance evaluated using the Area Under the Curve (AUC) and calibration analysis. Model interpretability was enhanced via SHapley Additive exPlanations (SHAP). **Results**: The Decision Tree model demonstrated the optimal balance between performance and interpretability. It achieved an AUC of 0.921 (95% CI: 0.848–0.988) in the internal validation set and maintained robust generalizability in the external SHARE cohort with an AUC of 0.958 (95% CI: 0.931–0.985). The nine key predictors identified were stroke history, BMI, HDL, loneliness, walking speed, disease duration, age, recall summary score, and total cholesterol. SHAP analysis visualized the specific contribution of these features to individual risk. **Conclusions**: This study successfully developed a high-performance, explainable, lightweight ML model for sarcopenia risk prediction. By inputting only nine readily available clinical indicators via an online tool, individualized risk assessment can be generated. This facilitates early identification and risk stratification of sarcopenia in older European arthritis patients, thereby providing valuable decision support for implementing precision interventions.

## 1. Introduction

Sarcopenia, a progressive skeletal muscle disorder characterized by the loss of muscle strength and mass, shares a complex relationship with arthritis [1]. Growing evidence indicates that this comorbidity significantly worsens clinical outcomes; specifically, sarcopenia can accelerate disease progression, compromise treatment efficacy, increase susceptibility to adverse drug reactions, and profoundly impair both functional capacity and quality of life [1,2].

A growing number of cross-sectional studies have recently investigated the determinants of sarcopenia in patients with arthritis. Demographic and socioeconomic factors serve as a fundamental basis for predicting sarcopenia in patients with arthritis. The key variables closely associated with sarcopenia in this population include sex, age, marital status, education level, and employment status [3,4,5]. Arthritis-related symptoms and comorbidities, such as disease duration, pain, physical function (including Instrumental Activities of Daily Living (IADL); Activities of Daily Living (ADL), and walking speed), fractures, and comorbidities, are significantly associated with the occurrence of sarcopenia in patients with arthritis [6,7,8,9]. Cognitive impairment is relatively common in older adults with arthritis and is closely associated with declines in physical function and the presence of sarcopenia [10]. Concurrently, diminished social participation and social isolation may exacerbate feelings of loneliness and helplessness in arthritis patients [11]. Depression and sleep disturbances, among other psychological and behavioral factors, play critical roles in the pathogenesis of sarcopenia in patients with arthritis [12,13]. The persistent discomfort and functional limitations caused by arthritis predispose patients to depressive symptoms. Chronic inflammation, dyslipidemia, anemia, and dysglycemia collectively form a core pathophysiological network that adversely impacts muscle mass and function [14].

Sarcopenia in patients with arthritis is not driven by a single factor but results from the complex interplay of multiple determinants. These factors are interconnected and collectively influence the sarcopenia status of arthritis patients.

In cross-sectional studies, traditional statistical methods have several limitations when dealing with complex data relationships and prediction tasks. For example, in the context of sarcopenia research among arthritis patients, the actual influencing factors often involve intricate nonlinear relationships. The traditional modeling process requires manual variable selection and feature extraction, which heavily rely on domain expertise and experience. However, traditional statistical methods face inherent limitations when addressing the complex, nonlinear interactions often observed in sarcopenia and arthritis research. These approaches typically rely on manual variable selection driven by domain expertise, which, while ensuring clinical plausibility, often assumes linear relationships and may inadvertently overlook novel predictors [15,16]. Furthermore, traditional models frequently struggle with high-dimensional datasets, limiting their ability to uncover latent patterns within large-scale longitudinal data and potentially compromising predictive accuracy.

In contrast, artificial intelligence has demonstrated immense potential in medical informatics, offering transformative solutions to these complex clinical challenges [17]. Machine learning (ML), a critical subset of artificial intelligence, can handle large datasets containing numerous predictor variables, effectively capture nonlinear relationships, uncover hidden patterns within massive amounts of data, and construct models that better reflect real-world scenarios, thereby improving predictive accuracy [18,19]. These advanced data analysis techniques are increasingly applied to decipher intricate biological mechanisms in high-dimensional settings [20]. Additionally, ML models can adaptively learn relevant features, resulting in strong generalization capabilities that allow them to adapt to sarcopenia prediction tasks in patients with arthritis across different settings [21].

Previous research on sarcopenia using machine learning (ML) has focused predominantly on community-dwelling older adults, while the development of predictive models specifically for elderly populations with arthritis remains notably inadequate. To date, only Wang et al. [22] have utilized ML methods to construct a risk prediction model for sarcopenia among older Chinese adults with knee osteoarthritis (KOA), making such explorations relatively rare. Wang et al. [22] developed and compared eight different machine learning prediction models to assess sarcopenia in a sample of 847 Chinese KOA patients aged over 65 years. Through least absolute shrinkage and selection operator (LASSO) regression and logistic regression analysis, they identified six key features strongly associated with sarcopenia in this population. Among all the ML models evaluated, the CatBoost algorithm demonstrated an exceptional area under the receiver operating characteristic curve (AUC) of 0.979 in the training set and maintained a high AUC of 0.9697 in the internal validation set, indicating robust predictive performance.

However, this study by Wang et al. [22] has several limitations. First, it focused exclusively on a Chinese KOA population, leaving a gap for ML-based explorations of sarcopenia in older European arthritis populations. Second, the study’s follow-up period was relatively short (only 4 years, utilizing data from the CHARLS 2011 and 2015 waves), and the sample size was limited (847 older Chinese adults with KOA). Finally, model validation was performed only internally via the same CHARLS database, and independent external validation was lacking. These factors constrain the generalizability and long-term stability of the developed ML model.

To address these research gaps, this study develops a machine learning-driven, explainable prediction model tailored for older European adults with arthritis. By leveraging the ELSA dataset for model training and incorporating independent external validation with the SHARE database, we enhance the generalizability of our personalized risk prediction framework. The utilization of longitudinal data spanning 12 years (2004–2016) enables the model to capture long-term sarcopenia progression trajectories, supporting dynamic risk assessment at the individual level. Furthermore, the integration of SHAP-based interpretability analysis and an interactive online risk calculator allows for transparent, clinically actionable insights.

This study aimed to develop and externally validate an explainable machine learning (ML) model to predict the risk of incident sarcopenia in older European adults with arthritis. The specific goal was to provide a practical, phenotype-based screening tool for primary care settings to facilitate the early identification of high-risk individuals and support risk-stratified clinical decision-making.

## 2. Materials and Methods

### 2.1. Study Design

**Figure 1:**
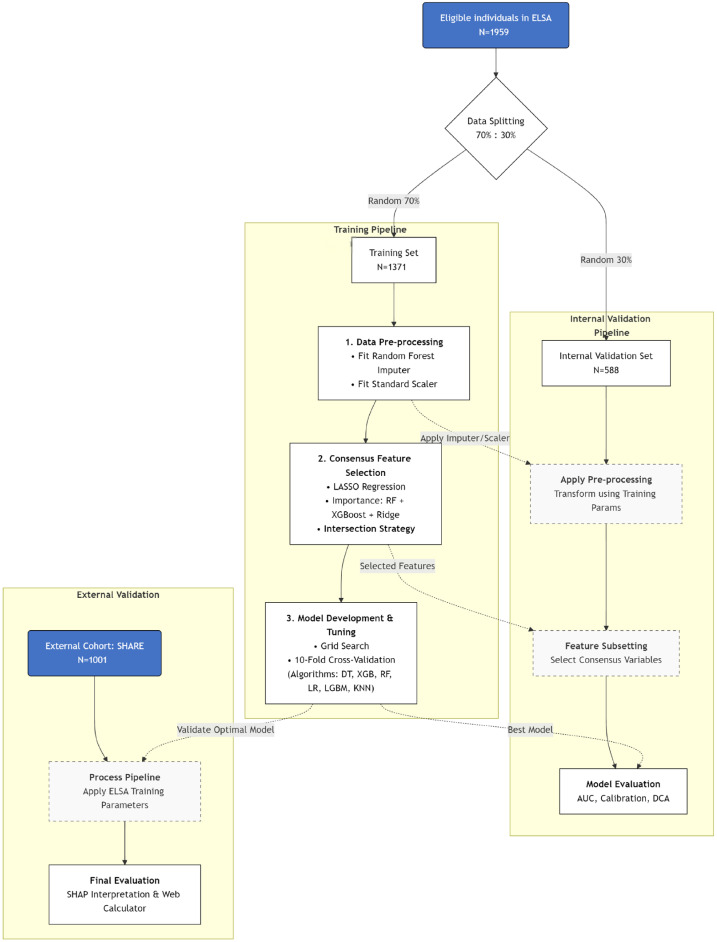
Flow diagram. Abbreviations: ELSA, English Longitudinal Study of Ageing; SHARE, Survey of Health, Ageing and Retirement in Europe; LASSO, Least Absolute Shrinkage and Selection Operator; AUC, Area Under the Curve; LR, Logistic Regression; RF, Random Forest; XGB, Extreme Gradient Boosting; DT, Decision Tree; KNN, K-Nearest Neighbors.

This research followed the TRIPOD checklist (Supplementary Table S1), with the study design detailed in Figure 1. Briefly, the ELSA database was used for model development and internal validation, and the SHARE database was used for external validation. These two data sources are prospective cohort studies that focus on older European populations and cover multidimensional variables related to health, socioeconomic status, and behavior, making them particularly suitable for joint investigations into complex health issues (detailed database descriptions are provided in the Supplementary Methods). We analyzed all available follow-up data from baseline to the most recent wave in both cohorts. To ensure temporal consistency across the databases, this study selected data from Wave 2 to Wave 8 of the ELSA database and from Wave 1 to Wave 6 of the SHARE database (with Wave 1 initiated in 2004), resulting in a uniform follow-up period of 12 years for both datasets.

### 2.2. Study Participants and Inclusion and Exclusion Criteria

(1) Inclusion criteria: ① age ≥ 65 years; ② diagnosed with arthritis at baseline; ③ not diagnosed with sarcopenia at cohort baseline. Arthritis status was ascertained based on participants’ self-reported physician diagnosis, strictly following the standardized procedures used in the ELSA and SHARE cohorts (question: ‘Have you ever been diagnosed with arthritis or rheumatism by a doctor?’). Although this operational definition encompasses a heterogeneous group of joint disorders, it represents a commonly used and validated approach in large-scale population-based research in this field [23,24].

Crucially, the utility of this definition for risk stratification has been supported by clinical rheumatology studies. For example, Ke et al. [25] applied the identical self-reported definition to predict cardiovascular risk and demonstrated that, despite the inability to differentiate osteoarthritis from rheumatoid arthritis, an overarching ‘arthritis phenotype’ remained a robust predictor of systemic outcomes Similarly, Ni et al. [26] confirmed the applicability of this approach for predicting pulmonary comorbidities and highlighted shared features across arthritis subtypes, particularly inflammatory burden and activity limitation, thereby supporting such population-level analyses. Consistent with these specialist rheumatology studies, our model is intended to provide a pragmatic sarcopenia screening tool for the heterogeneous arthritis population encountered in primary care, where initial risk assessment frequently precedes definitive rheumatologic subtyping.

(2) Exclusion criteria: ① missing data on arthritis diagnosis at baseline or during follow-up; ② missing data on components of the sarcopenia diagnostic criteria at baseline or during follow-up; ③ key variables in the variable screening with a missing value proportion exceeding 25% [27]. After applying the predefined inclusion and exclusion criteria, a total of 1959 participants from the ELSA dataset were ultimately included for machine learning modeling and internal validation, and 1001 participants from the SHARE dataset were used for external validation of the model. Figures S1 and S2 show the flowcharts of study participant inclusion and exclusion for the ELSA and SHARE databases, respectively.

### 2.3. Outcomes

Due to the absence of dual-energy X-ray absorptiometry (DXA) data in both the ELSA and SHARE cohorts, we were unable to utilize the DXA-based ‘gold standard’ for defining sarcopenia. Instead, we assessed sarcopenia based on a combination of grip strength and estimated skeletal muscle mass. This approach aligns with established methodologies in previous studies utilizing these cohorts; for instance, Ragusa et al. [28] and Veronese et al. [29] successfully employed the Lee anthropometric equation [30] as a reliable surrogate for muscle mass assessment in the ELSA cohort, and Qaisar et al. [31] applied a similar methodology in the SHARE cohort. The Lee anthropometric equation has been validated against DEXA in populations, demonstrating high predictive accuracy ($R^{2}$ = 0.86 in Lee et al. [30]) The detailed measurement protocols for handgrip strength, the equation for estimating skeletal muscle mass (SMM), and the calculation of the skeletal muscle mass index (SMI) are comprehensively described in the Supplementary Methods. The detailed evaluation criteria are also provided in the Supplementary Methods.

Our analysis of the measurement protocols revealed that both cohorts utilized the same dynamometer model (Smedley Dynamometer, TTM, Tokyo, Japan) and adhered to identical EWGSOP2 diagnostic thresholds (men < 27 kg, women < 16 kg). However, a methodological discrepancy exists in the measurement procedure: the ELSA cohort typically records the mean of three measurements, whereas the SHARE cohort records the maximum value derived from four measurements. While this procedural variation may introduce a degree of measurement heterogeneity, the model maintained robust performance in the external SHARE validation set. This suggests that the algorithm effectively captures the underlying biological phenotype of sarcopenia and demonstrates strong generalizability across diverse European measurement protocols.

### 2.4. Model Predictors

To strictly adhere to the TRIPOD-AI guidelines for prospective prediction modeling (predictors in Item 7a), we explicitly formalized the temporal architecture of our model to ensure its integrity as a prospective risk prediction tool rather than a mixed cross-sectional-longitudinal classifier (Figure S3). (1) Baseline assessment (2004): We defined the study baseline as the year 2004 (ELSA Wave 2; SHARE Wave 1). At this time point, all participants were screened, and those with prevalent sarcopenia were excluded. All predictor variables (sociodemographic, clinical, and biomarkers) were ascertained exclusively at the year 2004 (at the baseline assessment) and treated as time-fixed covariates. For example, the disease duration was strictly calculated as the time interval between the patient’s self-reported year of arthritis diagnosis and the baseline interview date (2004). Crucially, this calculation excluded the 12-year follow-up period, ensuring that the duration variable reflects only the information available at the time of screening (baseline assessment in 2004). Importantly, no information from subsequent waves (2006–2016), such as earliest available values, last observation carried forward, or cross-wave averaging, was incorporated into the predictor set. (2) Outcome Ascertainment Window (2006–2016): Surveillance for incident sarcopenia commenced at the first subsequent follow-up wave (Year 2006: Wave 3 for the ELSA and Wave 2 for the SHARE) and extended through 2016 (Wave 8 for the ELSA and Wave 6 for the SHARE). Incident sarcopenia was defined as a new diagnosis occurring strictly within this outcome ascertainment window among participants who were sarcopenia-free at the 2004 baseline (ELSA Wave 2; SHARE Wave 1).

Through the combination of a literature review (these literature-supported variables have been thoroughly reviewed in the Section 1) [3,4,5,6,7,8,9,10,11,12,13] and clinical expert evaluation, this study identified 33 potential variables (details are provided in the Supplementary Method section). These predictors provide a solid foundation for subsequently building an ML-based prediction model for sarcopenia in patients diagnosed with arthritis, holding promise for accurate prediction and early intervention of sarcopenia risk in arthritis patients.

In the field of machine learning, feature selection becomes a critical step for enhancing model performance and interpretability when dealing with multiple variables. After the 33 potential variables were obtained, this study further screened them on the basis of the principle of variable refinement to decide whether to include them in the final model [32].

All feature selection procedures (LASSO regularization parameter and variable-importance ranking based on Random Forest, XGBoost, and ridge regression models) were executed exclusively on the training dataset (N = 1371) after the initial 7:3 data splitting. The validation and external test sets were strictly withheld from this process to prevent data leakage. The consensus features identified in the training phase were subsequently extracted from the validation/test sets for model evaluation.

LASSO regression is a method for variable selection and regularization in regression analysis. Its advantage lies in its ability to perform variable selection automatically while estimating linear regression model parameters. By adding an L1 regularization term to the objective function, some coefficient estimates are forced to shrink to zero, thereby achieving variable selection. In high-dimensional data scenarios, LASSO regression can effectively reduce model complexity, avoid overfitting, and improve the model’s generalization ability [33].

We employed a ‘Consensus Feature Selection’ strategy by taking the intersection of top features identified by four distinct algorithms: LASSO Regression, Random Forest (RF) Regressor, XGBoost Classifier, and Ridge Regressor. This multi-model approach was chosen to leverage the complementary strengths of each algorithm: I. LASSO & Ridge: These linear models are highly effective at handling multicollinearity and providing regularization to prevent overfitting, but they assume linear relationships between predictors and the outcome. II. Random Forest & XGBoost: These tree-based ensemble methods excel at capturing complex non-linear interactions and high-order dependencies that linear models might miss.

By selecting only the features consistently ranked as important by both linear (Ridge) and non-linear (RF/XGBoost) algorithms, we filter out algorithm-specific artifacts and identify the most biologically robust predictors. This intersection approach ensures that the final feature set is stable and generalizable across different modeling assumptions. This rigorous consensus strategy aligns with recent clinical ML prediction studies [34].

To further evaluate the robustness of the identified features and rule out stability artifacts caused by data dependence, we conducted a stability analysis via a resampling strategy. We performed 10-fold cross-validation on the training set. In each iteration, the consensus feature selection process was repeated, and the selection frequency of each predictor was recorded. Features with a selection frequency of ≥80% across the folds were considered to exhibit high stability, indicating that they are consistent predictors rather than artifacts of specific data partitions.

Finally, the top 12 variables from each model were extracted, and the most critical core variables, which were consistently identified by all the models, were determined by taking the intersection of their Venn diagrams [34].

### 2.5. Modeling Pipeline: From Data Splitting to Optimization

Before any preprocessing (e.g., data imputation or feature scaling), we split the ELSA dataset into a training set (N = 1371; 70%) and an internal validation set (N = 588; 30%). The SHARE dataset (N = 1001) was reserved as a fully independent external test cohort. This workflow is consistent with the TRIPOD + AI recommendations [35], which emphasize that performing data imputation or feature scaling on the entire dataset prior to data splitting can lead to data leakage and systematic overestimation of model performance.

We first excluded variables with >25% missingness. The choice of a 25% threshold is consistent with rigorous machine-learning research using the same ELSA cohort. Specifically, Leme et al. [36] developed machine-learning models to predict frailty and applied a stringent missing-data strategy; Random Forest (RF) imputation was used for predictor variables with <25% missingness. From a statistical standpoint, although RF imputation is generally robust, its reliability decreases as the proportion of missing information becomes excessive. Dong et al. [37] noted that even with advanced imputation approaches, missingness above 25% may reduce statistical power and introduce bias. Therefore, we also removed participants with >25% missing data to balance sample-size retention against imputation quality. While RF-based imputers are resilient, including participants with extensive missingness would force the model to rely heavily on imputed values, potentially introducing noise that could outweigh the benefit of a larger sample size [27]. Thus, the 25% cutoff aligns with common methodological recommendations in clinical research to ensure analytic stability.

In studies by Leme et al. [36], which constructed machine learning models for elderly sarcopenia patients in UK communities, the random forest (RF) method was used for imputation of missing values in predictor variables with a missing proportion of less than 25%. When data dimensionality is high, variable types are mixed (including numerical and categorical variables), the proportion of missing data is moderate, and no linear relationship between variables needs to be assumed. The random forest (RF) imputation method can provide high-precision, low-bias imputations while maintaining the integrity of the data structure.

Similarly, in our machine-learning screening study of European arthritis patients with sarcopenia, missing values in the remaining variables were imputed using an RF imputer fitted exclusively on the training set. This design ensured that distributional information from the internal validation set or the external test cohort did not influence the imputation process, thereby preventing leakage. The trained imputer was then applied to the internal validation set and the external SHARE cohort. The observed missing proportions were 4.791% for stroke, 13.885% for BMI, and 13.68% for the recall summary score. Diagnostic plots (Supplementary Table S2) indicated that the probability density distributions of key variables (e.g., stroke, BMI, and recall summary score) were consistent before and after imputation, suggesting that imputation did not materially alter the distributional structure. In addition, standardization parameters were derived only from the training set and then applied to the internal validation set and the external SHARE cohort.

To prevent data leakage, all feature selection procedures were performed after the initial data splitting. All feature selection procedures were performed using the training data only. This included selection of the LASSO regularization parameter and variable-importance ranking based on Random Forest, XGBoost, and ridge regression models. The core predictors identified during training were subsequently used to extract features in the validation dataset. Under this workflow, neither the validation set nor the test cohort contributed to model training or feature selection, thereby preventing data leakage at the design level.

Six ML algorithms (XGBoost, logistic, random forest, decision tree, KNN, and LightGBM) were subsequently employed to develop the prediction models via the training data. Rather than using default settings, we optimized all six machine-learning algorithms using grid search combined with 10-fold cross-validation. Importantly, cross-validation was conducted exclusively within the training set and was not applied to the internal validation set (N = 588). We added Supplementary Table S5 to report the full set of final hyperparameters. For example, to mitigate overfitting, the selected Decision Tree model constrained max_depth to 20 and set min_samples_split to 50. For XGBoost, we tuned key parameters including learning_rate = 0.1 and max_depth = 8. These details support full reproducibility of model development and performance.

The optimal model, which was selected from the internal validation set, underwent a thorough evaluation based on multiple performance metrics: (1) AUC: AUC values ranging from 0 to 1 and values closer to 1 indicate stronger discriminatory power; (2) specificity: high specificity in sarcopenia risk prediction means that the model can accurately identify individuals without sarcopenia risk, reducing misclassification as frail; (3) precision: high precision in the context of sarcopenia risk prediction indicates that among individuals predicted by the model to be at risk of sarcopenia, the proportion truly at risk is high; (4) sensitivity: high sensitivity in sarcopenia risk prediction means that the model can detect as many individuals at risk of sarcopenia as possible, reducing missed diagnoses; (5) F1 score: the F1 score, providing a balanced measure of precision and sensitivity; (6) decision curve analysis (DCA) plots: DCA assesses the clinical net benefit across various risk thresholds; (7) precision–recall (PR) curves: PR curves complement the ROC analysis by illustrating the trade-off between precision and recall [38].

After the optimal model was determined, 10-fold cross-validation was implemented on the training set to improve its generalizability and robustness. The training cohort was randomly split into 10 subsets. Each time, 9 subsets were used as training data, and 1 subset was used as validation data; the process was repeated 10 times for training and validation [39].

To further validate the model’s generalizability, an external validation was conducted using the independent SHARE dataset. The model was evaluated on this external set via the same metrics (AUC, specificity, precision, sensitivity, F1 score, DCA, PR curves, etc.) as those used in the internal validation phase. To assess the external transportability and generalizability of the model to the SHARE cohort, we evaluated the calibration performance via the calibration slope and intercept. Given the potential systematic bias introduced by heterogeneity in grip strength measurement protocols (mean values in ELSA vs. maximum values in SHARE), we did consider and perform post hoc logistic recalibration (intercept and slope adjustment) in the external cohort as a sensitivity analysis. This procedure corrected the intercept to 0.00 and the slope to 1.00, effectively removing the systematic bias caused by the protocol differences. The overall improvement in predictive accuracy was quantified by the change in the Brier score, a composite measure of discrimination and calibration.

Moreover, SHAP was used to provide explanations for the optimal model and to rank the importance of features. By calculating the SHAP value for each feature, it is possible to intuitively determine which features have a greater impact on the prediction of sarcopenia risk in elderly European arthritis patients. Finally, an online risk prediction model calculator was developed. Further methodological details are described in the Supplementary Methods.

## 3. Results

### 3.1. Population Characteristics

Figure S1 illustrates the screening process for the ELSA dataset. Finally, 1959 participants included in the machine learning analysis. The demographic and clinical characteristics of the study participants (n = 1959) are detailed in Supplementary Table S3, with data presented separately for the training (n = 1358) and internal validation (n = 583) sets. As shown in Supplementary Table S3, there were no clinically relevant differences in key demographic or clinical variables between the two sets (all *p* > 0.05).

For clarity and conciseness, Table 1 focuses on the demographic characteristics and the nine core predictors identified through feature selection. To further assess the distributional balance beyond *p* values, we calculated standardized mean differences (SMDs). We emphasized SMDs over *p* values for this assessment because *p* values are highly sensitive to large sample sizes and may indicate statistical significance even for clinically negligible differences. In contrast, SMDs provide a robust measure of the magnitude of imbalance independent of sample size. As shown in Table 1, the SMDs for all core predictors, including BMI, walking speed, loneliness, and biochemical markers, were consistently below 0.1 (ranging from <0.001 to 0.084). This negligible difference confirms that the data splitting process did not introduce selection bias.

Figure S2 shows the screening process for the SHARE dataset. A total of 1001 participants were ultimately included for external validation via machine learning.

### 3.2. Variable Screening

**Figure 2:**
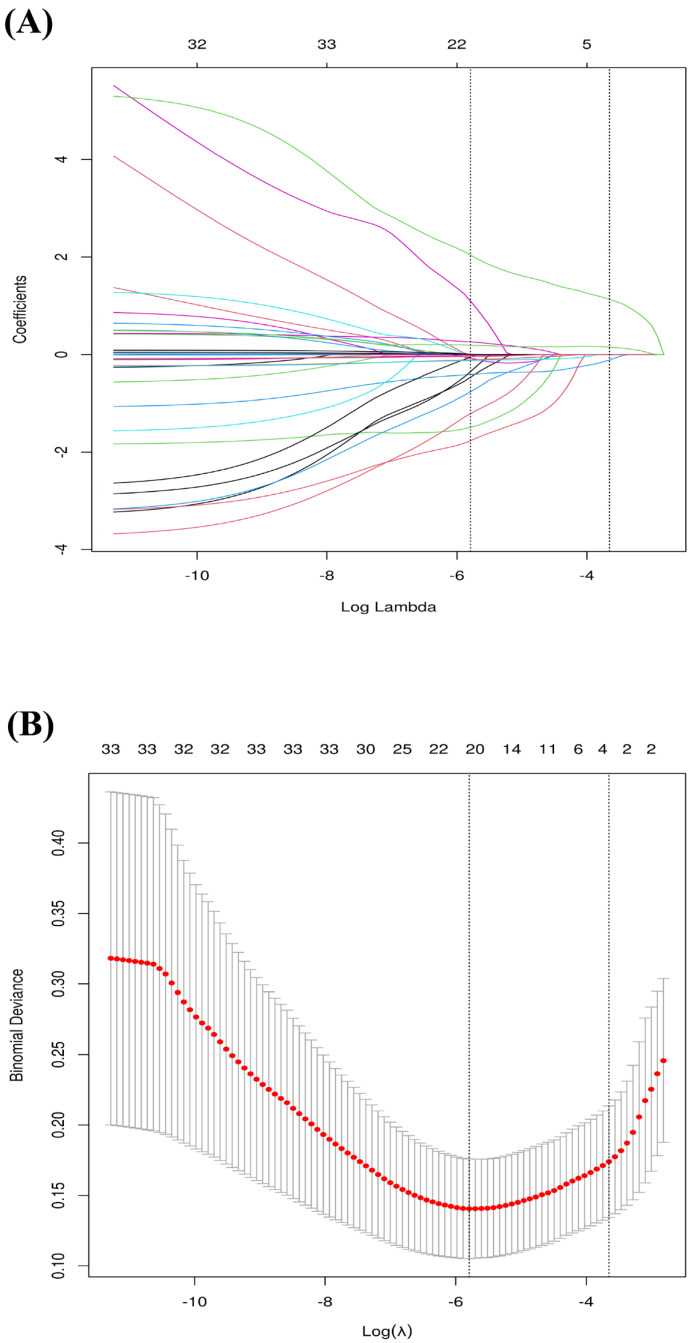
Feature selection using the LASSO regression model. (**A**) LASSO coefficient profiles of the candidate variables. Each curve corresponds to a variable. (**B**) Selection of the optimal penalization coefficient (λ) in the LASSO model using 10-fold cross-validation. Abbreviations: LASSO, Least Absolute Shrinkage and Selection Operator.

**Figure 3:**
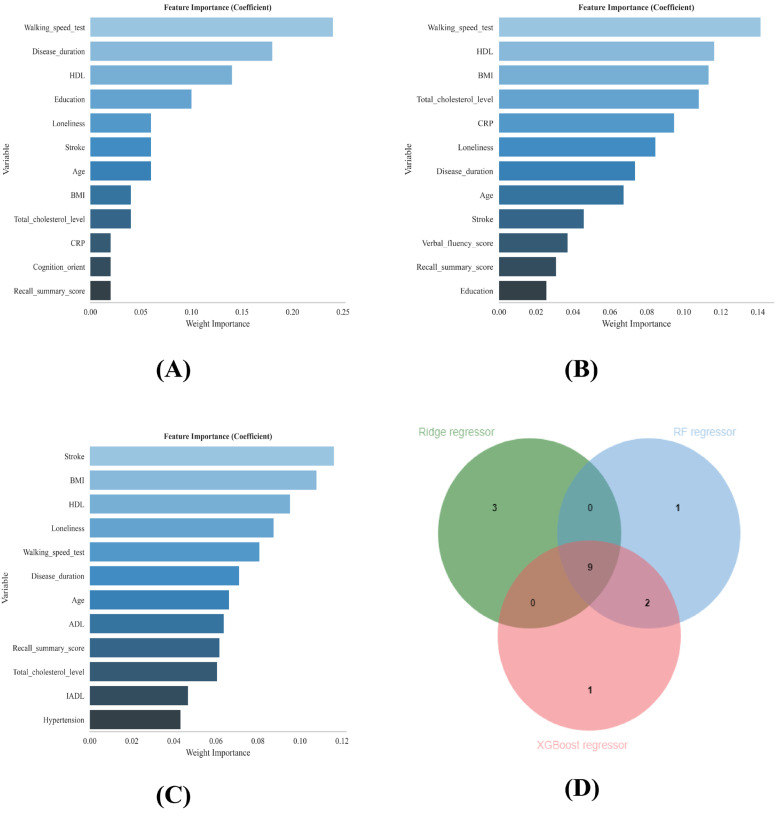
(**A**–**C**) Ridge regressor, RF regressor and XGBoost regressor were used to analyze the variable importance ranking. (**D**) Venn diagram illustrating the intersection of top predictors across methods, identifying nine consensus features for final model inclusion. RF: Random forest; XGBoost: extreme gradient boosting.

LASSO regression was used to select 19 features significantly associated with sarcopenia in patients with arthritis by adjusting the regularization parameter λ (Figure 2A,B). To identify the most robust predictors, variable importance was assessed via three distinct machine learning algorithms: ridge regression, XGBoost regression, and random forest (RF) regression. The top 12 features from each model’s ranking were compared, and their common subset was determined by taking the intersection via Venn diagrams (Figure 3A–C). These intersecting features include stroke, BMI, HDL, loneliness, walking_speed_test, disease_duration, age, recall_summary_score, and total_cholesterol_level. These common variables are used for subsequent machine learning modeling (Figure 3D).

The stability analysis confirmed the robustness of these nine core predictors across data perturbations. As detailed in Supplementary Table S4, seven of the nine predictors, specifically age, BMI, HDL, loneliness, walking speed, disease duration, and total cholesterol, achieved a selection frequency of 100% (selected 10/10 folds). The remaining two predictors, the stroke and recall summary scores, demonstrated high stability, appearing in 80% (8 out of 10) of the cross-validation folds. This high frequency confirms that these selected variables are true indicators of robustness rather than artifacts of specific data partitions.

### 3.3. Model Development and Performance Comparison

**Figure 4:**
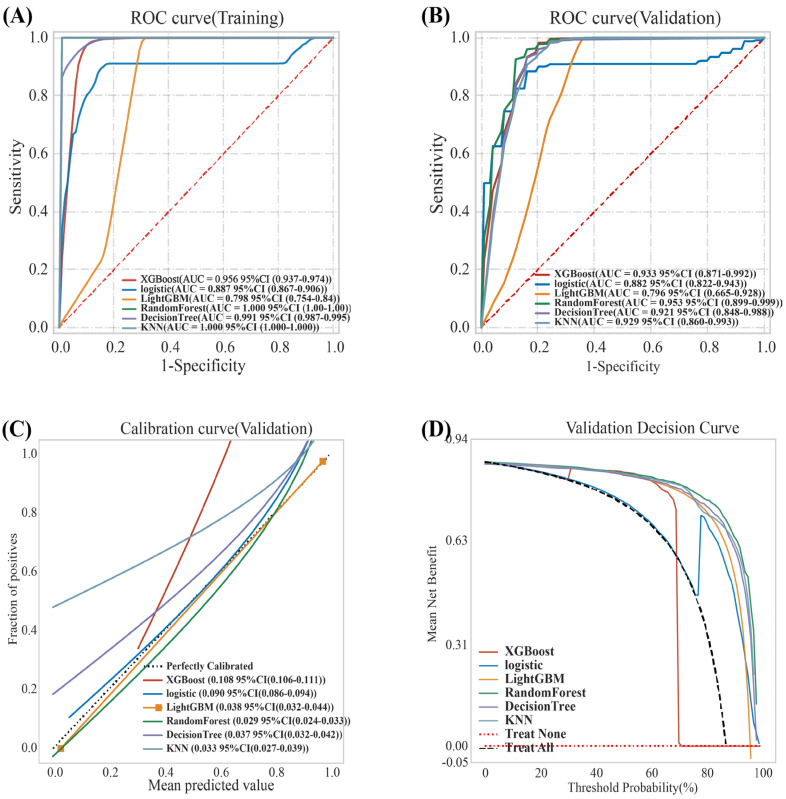
Receiver operating characteristic curve performance of the six models on the training (**A**) and validation (**B**) sets; (**C**) Calibration curve. Calibration curves of the machine learning models in the internal validation set. The x-axis represents the predicted probability of sarcopenia, and the y-axis represents the actual observed proportion. The dashed diagonal gray line indicates perfect calibration. The Brier score for each model is displayed within the plot area. To provide a complete assessment of calibration performance, the calibration intercept and slope for each model are reported as follows: Random Forest (−0.059, 1.005), Decision Tree (0.144, 0.889), Logistic Regression (0.213, 0.819), XGBoost (−0.472, 0.868), LightGBM (−0.418, 0.503), and KNN (0.985, 0.100). Ideally, a perfectly calibrated model has an intercept of 0 and a slope of 1 (**D**) Decision curve analysis evaluating the clinical net benefit of each model across different threshold probabilities. The ‘ALL’ curve assumes all patients are positive, while the ‘NONE’ curve assumes none are positive.

Random forest and KNN achieved an AUC = 1.0 in the training set, indicating severe overfitting (Figure 4A).

In the training set, both the Random Forest and KNN algorithms achieved perfect discrimination with an AUC of 1.000 (Table 2). However, rather than indicating predictive excellence, these perfect scores were identified as indicators of severe overfitting. To ensure clinical reliability, model selection was governed exclusively by performance in the internal validation set. Consequently, despite their high training scores, the Random Forest and KNN models were deemed methodologically unsuitable for clinical prediction due to their lack of generalizability.

LightGBM had the lowest AUC values in the internal validation set [AUC = 0.796 (0.665–0.928)], indicating substandard performance and significant underfitting. Compared with XGBoost, logistic [0.882 (95% CI: 0.822–0.943)] had significantly lower AUC values [0.933 (95% CI: (0.871–0.992)] (Table 2 and Figure 4B).

Although the XGBoost model demonstrated a marginal advantage in discrimination, achieving a slightly higher AUC (0.933 vs. 0.921), precision (0.936 vs. 0.910), and sensitivity (0.951 vs. 0.930) compared to the Decision Tree (Table 2), it was disqualified due to two critical failures in clinical reliability, as detailed in our results:

**①** Poor Calibration: The XGBoost calibration curve deviated substantially from the ideal diagonal (red solid line, Figure 4C), resulting in a significantly higher Brier score (0.108) compared to the Decision Tree (0.032). Furthermore, the XGBoost model exhibited a problematic calibration intercept of −0.472, indicating a systematic bias towards risk overestimation, whereas the Decision Tree’s intercept of 0.144 was much closer to the ideal value of 0. Importantly, the primary objective of this study was to provide a clinically reliable screening tool for primary care settings. In this context, well-calibrated risk probabilities, ensuring that a predicted risk level accurately reflects the true event rate, are more critical for guiding appropriate patient referrals than maximal discrimination (AUC) alone. Consequently, despite the marginal AUC advantage of XGBoost, it was deemed unsuitable because of its potential to mislead clinical decision-making.

**②** Lack of Clinical Net Benefit: Decision Curve Analysis (DCA) revealed that the XGBoost model provided a net benefit lower than the ‘treat-none’ baseline across key threshold probabilities, rendering it clinically impractical (Figure 4D).

Therefore, on the basis of a comprehensive analysis, the decision tree demonstrated the best overall predictive performance among the six machine learning models and was selected for subsequent analysis. Additionally, the parameter values are summarized in Supplementary Table S5.

### 3.4. Model Stability

**Figure 5:**
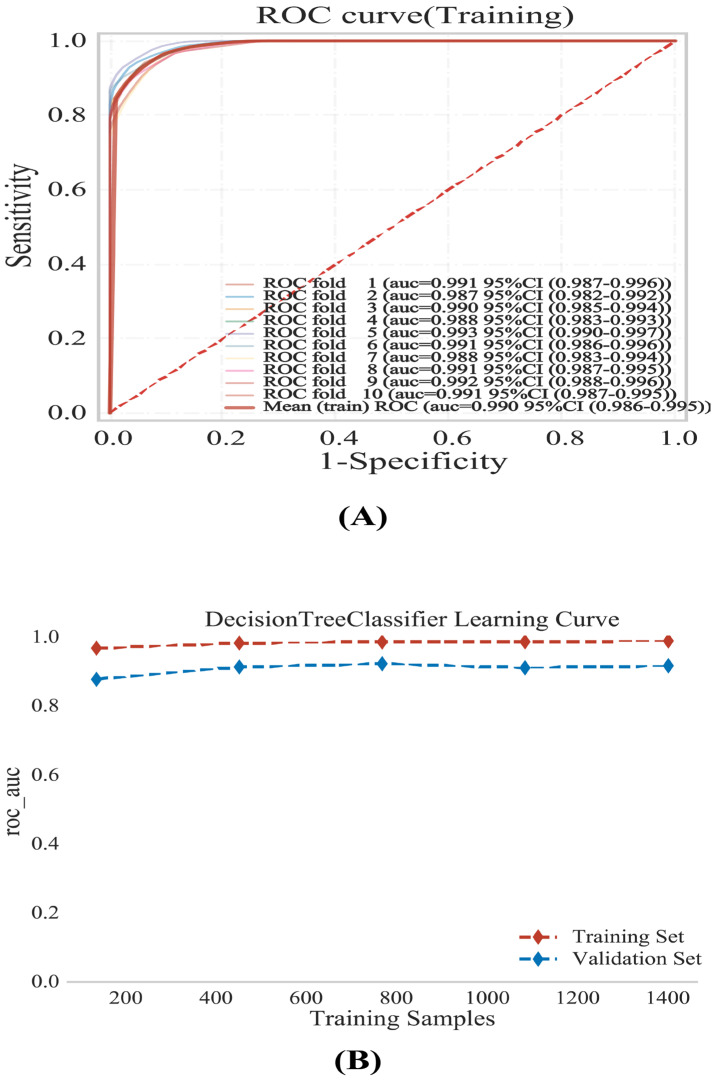
Development and internal validation of the optimal Decision Tree model using 10-fold cross-validation. ROC curves demonstrate model performance on the (**A**) training, (**B**) validation.

Following the selection of the Decision Tree as the optimal algorithm, we conducted a rigorous assessment of its stability and learning behavior exclusively within the training set (*N* = 1371). First, we performed 10-fold cross-validation exclusively within the training set to quantify the stability of discrimination across different resampled partitions. As shown by the fold-specific ROC curves and their mean ROC (Figure 5A), the model exhibited consistently high AUC values across folds (mean training AUC = 0.990, 95% CI: 0.986–0.995), indicating robust performance within the training data. To further examine whether the model was adequately trained and to assess potential overfitting, we generated a learning curve by progressively increasing the training sample size and tracking the AUC on the training subset and the corresponding validation subset (Figure 5B). The learning curve shows that the AUC values stabilized as the sample size increased, supporting good model fit and sufficient training.

### 3.5. External Validation of the Decision Tree Model

Variables in SHARE were mapped to align with ELSA definitions. A Random Forest imputer was fitted using only the ELSA training data. This pre-trained imputer was then applied to predict missing values in the SHARE dataset. Feature scaling was calculated exclusively from the ELSA training set and applied to the SHARE cohort.

The baseline characteristics of the SHARE cohort (N = 1001) are summarized and compared with the ELSA training cohort (1371) and ELSA internal validation cohort (N = 588) in Supplementary Table S6. While the ELSA and SHARE cohorts shared comparable distributions in sex (females: 50.1% vs. 54.1%) and sarcopenia prevalence (12.9% vs. 15.1%), significant heterogeneity was observed in some sociodemographic and psychosocial profiles. For instance, the prevalence of living without a spouse was notably higher in the SHARE cohort compared to ELSA (27.8% vs. 6.5%), and the SHARE population reported higher levels of loneliness (median score: 3.00 vs. 1.25). Additionally, the SHARE cohort had a lower median BMI (25.2 vs. 27.4 kg/m^2^) and a distinct lifestyle profile (e.g., lower smoking rates). Despite these population heterogeneities, the consistency in model performance underscores the robustness of the identified predictors.

The Decision Tree prediction model demonstrated exceptional performance in the external validation cohort (SHARE database), achieving an AUC of 0.958 (95% CI: 0.931–0.984) alongside balanced sensitivity (0.948) and precision (0.933).

With respect to model calibration, the original decision tree model exhibited a calibration intercept of −0.899 and a slope of 1.175 in the SHARE cohort (Figure S4). The negative intercept reflects a systematic overestimation of sarcopenia risk, which is likely attributable to the higher thresholds for positive cases in SHARE owing to the use of maximum grip strength values. After applying logistic recalibration, the calibration parameters were successfully optimized (Intercept = 4.468 × 10^−16^ ≈ 0; Slope = 1.000 × 10^0^ ≈ 1), indicating a near-perfect alignment between the predicted and observed probabilities. Consequently, the Brier score was reduced from 0.0390 to 0.0289. This reduction in the Brier score signifies that recalibration effectively minimized the error of the probabilistic predictions, resulting in a more reliable tool for absolute risk estimation in diverse clinical settings.

Furthermore, decision curve analysis (DCA) validated the model’s clinical utility, showing a clear net benefit over default strategies. Collectively, these findings position the Decision Tree model as a viable and effective tool for sarcopenia screening among older adults with arthritis in EU countries (Figure S4).

### 3.6. Model Interpretation

**Figure 6:**
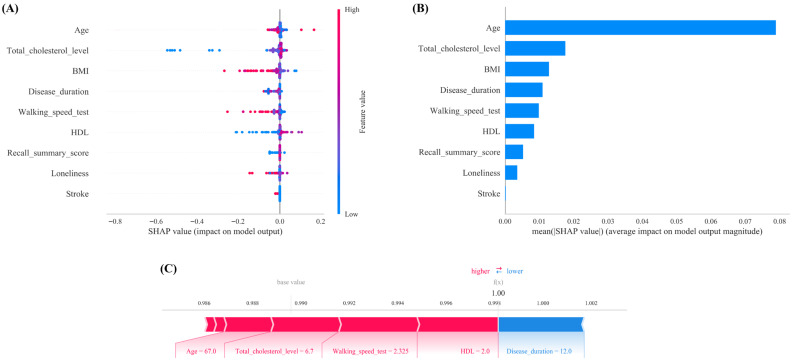
(**A**) SHAP interprets the model. (**B**) Feature importance ranking as indicated by SHAP. (**C**) Individual efforts by European elderly arthritis participants with sarcopenia.

SHAP analysis provided transparent insights into the Decision Tree model’s decision-making process (Figure 6). The summary plots (Figure 6A,B) identify age, total cholesterol, BMI, disease duration, walking speed, and HDL as the top contributors to sarcopenia risk.

Higher values of age, total cholesterol, and disease duration (indicated by red dots in Figure 6A) consistently drive the model’s prediction toward high risk. In contrast, for BMI and walking speed, it is the lower values (blue dots) that are strongly associated with positive SHAP values (Figure 6B).

Clinically, this implies that the phenotype of sarcopenia in arthritis patients is characterized by the coexistence of low body mass, reduced mobility, and metabolic abnormalities. Identifying this specific constellation of signs should trigger immediate comprehensive sarcopenia assessment in primary care settings.

Figure 6C presents a representative high-risk case analysis, where specific features, including Age = 67, Total Cholesterol = 6.7, Walking Speed = 2.325, and HDL = 2.0, were identified as the primary contributors driving the model’s risk estimation for this individual (large red bars). By quantifying feature contributions, SHAP analysis improves the transparency of the machine learning model, offering clinicians insights into which patient characteristics most strongly influence the risk stratification.

Finally, we developed an online calculator (https://www.xsmartanalysis.com/model/list/predict/model/html?mid=29187&symbol=8kL176161974Eezv62mC, accessed on 8 November 2025). (Figure 6). For the sake of transparency and reproducibility, we confirm that this online tool incorporates the fully trained and optimized Decision Tree model, with the exact hyperparameter configurations (Supplementary Table S5) used in the validation phases. No post hoc simplification was applied, ensuring that the tool’s predictive ability strictly aligns with the reported performance metrics. Healthcare providers in rheumatology and immunology departments can use this tool to predict the risk of sarcopenia in older European adults with arthritis by inputting 9 easily obtainable clinical variables.

## 4. Discussion

### 4.1. Principal Results

On the basis of the ELSA database and the SHARE cohort for external validation, this study developed and validated an interpretable machine learning model to predict the risk of sarcopenia in older European adults with arthritis. By integrating the results of LASSO regression and variable importance rankings, nine core predictors were selected from 33 initial variables (Figure 2 and Figure 3). A comparison of six machine learning algorithms demonstrated that the decision tree model achieved the best overall performance: on the internal validation set, the area under the curve (AUC) was 0.921 (95% CI: 0.848–0.988), with a sensitivity of 0.928, specificity of 0.838, and an F1 score of 0.950 (Table 2 and Figure 4). External validation on the SHARE cohort yielded an AUC of 0.958 (95% CI: 0.931–0.985), confirming the model’s robust generalizability across populations (Figure S4). SHAP interpretability analysis further elucidated the driving mechanisms behind sarcopenia risk in arthritis patients, identifying the following core risk factors: stroke, BMI, HDL, loneliness, walking speed test, disease duration, age, recall summary score, and total cholesterol level (Figure 5). On the basis of these findings, an online risk prediction tool for sarcopenia in arthritis patients was developed. This tool allows healthcare professionals to generate an individualized probability of sarcopenia by inputting these nine simple clinical measures, thereby providing decision support for the early intervention of sarcopenia in community and rheumatology settings (Figure 6). Notably, the etiologic drivers of inflammation-mediated diseases are not necessarily identical across conditions. For example, osteoarthritis (OA) is primarily initiated by mechanical loading and structural joint degeneration, whereas rheumatoid arthritis (RA) is primarily driven by autoimmune-mediated inflammation. However, Tournadre et al. [40] have shown that OA and RA share, at least in part, overlapping downstream pathways leading to sarcopenia, with pain-related disuse atrophy constituting a major driver of muscle loss in both diseases. Therefore, when modeling sarcopenia as the study outcome, we did not differentiate specific upstream subtypes of inflammation-mediated disease.

### 4.2. ML Method Selection

Traditional linear models typically assume that relationships among variables are fixed (e.g., linear and additive) across the entire population. In contrast, machine-learning approaches, particularly the decision tree model identified as optimal in our study, are well suited to capturing nonlinear patterns in complex and heterogeneous populations. Decision trees can automatically partition the sample into distinct subgroups via data-driven split points, enabling accurate outcome prediction even without relying on definitive diagnostic subtype labels.

Accordingly, regardless of which inflammatory arthritis subtype a patient has (e.g., OA or RA), if they exhibit the clinically observable phenotypic features identified through feature selection, such as stroke history, BMI, HDL, loneliness, walking speed test performance, disease duration, age, recall summary score, and total cholesterol level, the model can flag elevated risk of sarcopenia. In other words, the model primarily learns clinical phenotypes rather than inferring risk solely from presumed etiology.

In a previous systematic review, Leghissa M et al. [41] examined ML algorithms for sarcopenia in elderly individuals and reported the following as the most commonly used algorithms: random forest, LightGBM, logistic regression, XGBoost, decision tree and k nearest neighbors.

Logistic regression and decision tree models are simple and interpretable, allowing rheumatology healthcare providers to easily understand how the model can predict sarcopenia on the basis of patient characteristics, which is crucial for clinical decision-making. Additionally, these ML models have relatively low data requirements, can handle various data types, can operate quickly, and align well with the multifaceted nature of arthritis patient data.

The random forest algorithm offers strong resistance to overfitting, enabling the model to maintain good stability and generalizability when dealing with complex sarcopenia data in arthritis patients. Moreover, it can handle high-dimensional data without strict assumptions about data distribution, accommodating various potential distributions in arthritis sarcopenia data.

The LightGBM algorithm is highly computationally efficient and capable of processing large volumes of arthritis sarcopenia data in a short time. It also has low memory usage when handling large datasets and excels in accuracy. Through its histogram-based algorithm and leafwise growth strategy, the LightGBM can better capture complex patterns in the data, improving the prediction accuracy for arthritis-related sarcopenia.

XGBoost has strong generalization ability and can handle complex nonlinear relationships. Sarcopenia in arthritis patients often involves intricate interactions among multiple factors, and XGBoost can effectively capture these relationships. It is also computationally efficient and can quickly process large-scale arthritis patient data.

The KNN algorithm does not require pretraining, making it advantageous for complex and highly individualized conditions such as arthritis and sarcopenia. It adapts well to local data characteristics and can provide accurate predictions even with complex data distributions. It also exhibits certain robustness to noisy data.

### 4.3. Comparative Analysis with the Previous ML Model Study

Previously, Wang et al. [22] developed eight machine learning (ML) prediction models for screening for sarcopenia in Chinese patients aged 65 years and above with knee osteoarthritis (KOA). Their data were sourced from the CHARLS database covering the period from 2011–2015. After applying the inclusion and exclusion criteria, a total of 847 KOA patients were included. These patients were divided into a training set and an internal validation set at a 7:3 ratio. Using LASSO regression and logistic regression analysis, Wang et al. [22] identified six features closely associated with sarcopenia in patients with KOA. They constructed ML models for KOA-associated sarcopenia via eight algorithms. Among these, the CatBoost model demonstrated the best performance: on the internal validation set, it achieved an area under the ROC curve (AUC) of 0.969, an accuracy of 0.8902, and a Brier score of 0.0691. Wang et al. [22] subsequently enhanced the interpretability of the model via SHapley Additive exPlanations (SHAP) and developed an online prediction calculator to improve the clinical utility of the optimal model.

However, this study has several limitations. First, the research focused solely on a specific subgroup of individuals with KOA combined with sarcopenia, which limits its generalizability to other arthritis populations with sarcopenia. Furthermore, the study included only older Chinese adults, without involving elderly populations of other ethnicities. Thus, the model’s applicability for screening for sarcopenia in European populations with arthritis remains unknown. Second, the study relied solely on short-term follow-up data (2011–2015) from the CHARLS database. Consequently, the model’s stability for long-term sarcopenia risk prediction (e.g., over a follow-up period exceeding 10 years) remains uncertain. Third, the model was developed and validated via only split-sample internal validation from the CHARLS database, and independent external validation with cohorts from other regions is lacking. This reliance on internal validation from a single source carries a risk of overfitting, and the model’s performance may degrade when it is applied to other independent populations. Finally, although the initial population consisted of 30,380 individuals, only 847 participants were included after applying the inclusion and exclusion criteria for model construction and validation. This relatively small sample size may compromise the model’s stability and generalizability.

In contrast to prior research, such as the study by Wang et al. [22], our work distinguishes itself through three critical advancements: broader population scope, rigorous external validation, and enhanced clinical interpretability. First, regarding population, while Wang et al. [22] focused exclusively on a single-center cohort of Chinese patients with knee osteoarthritis, the present research incorporated two major prospective cohorts, ELSA (UK) and SHARE (multiple European countries), thereby extending the study population to older arthritis patients from major European nations such as the UK, Germany, France, Italy, and Spain. This provides a more universal tool for screening for sarcopenia in older European arthritis patients. Second, unlike Wang et al.’s study [22], which relied solely on internal validation, our study included the SHARE cohort data as an independent external validation set, mitigating the risk of overfitting associated with the use of homologous data. Finally, we prioritized interpretability to address the ‘black-box’ limitations of complex machine learning algorithms. By selecting the Decision Tree model, which offers transparent decision rules, and integrating SHAP analysis to visualize individual risk drivers, our approach bridges the gap between algorithmic performance and clinical trust, facilitation practical adoption in primary care settings.

### 4.4. Impact of Measurement Heterogeneity and Model Recalibration

A critical consideration in our external validation is the heterogeneity in grip strength assessment protocols. According to the EWGSOP2 guidelines, sarcopenia diagnosis relies on fixed cutoff points for continuous variables (e.g., low handgrip strength: <27 kg for men and <16 kg for women). Importantly, handgrip strength was assessed via different protocols across cohorts: ELSA used the mean of three trials, whereas SHARE used the maximum of four trials. Shifting from a mean to a maximum protocol systematically shifts the grip strength distribution rightward relative to the fixed diagnostic threshold, thereby altering cohort-level prevalence.

Prior work by Lim et al. [42] demonstrated that the maximum grip strength (HGSmax) is, on average, greater than the mean grip strength (HGSave) (approximately 2.3 kg; 24.01 kg vs. 21.72 kg). Around diagnostic thresholds, such a difference is sufficient to reclassify a substantial proportion of borderline individuals from sarcopenia to non-sarcopenia, leading to a markedly lower estimated prevalence (33.0% under HGSave vs. 19.5% under HGSmax) and only moderate agreement between definitions (Kappa = 0.604) [42]. Consistent with this mechanism, because SHARE adopts HGSmax, fewer participants fall below the EWGSOP2 cutoff points, resulting in fewer positive cases than would be expected under an HGSave protocol.

This theoretical expectation is consistent with our empirical calibration results in the SHARE cohort. The calibration intercept was negative (Intercept = −0.899), and the original calibration curve was situated below the ideal diagonal (Figure S4), confirming that the model systematically overestimated the absolute risk of sarcopenia in the external cohort due to the disparity in the protocol [43].

To address this systematic bias explicitly, we performed model recalibration (intercept and slope adjustment) for the external cohort. As expected, recalibration aligned the calibration parameters to their ideal targets (Intercept = 4.468 × 10^−16^ ≈ 0; Slope = 1.000 × 10^0^ ≈ 1). Importantly, the Brier score improved from 0.0390 to 0.0289 following adjustment. This improvement suggests that while the systematic bias introduced by protocol heterogeneity affects absolute risk estimates, the core predictors identified by our decision tree model are robust and generalizable. For clinical implementation in settings utilizing maximum grip strength protocols, our findings recommend a simple intercept adjustment to ensure precise individual risk stratification.

### 4.5. Interpretation of the SHAP Results

Previous cross-sectional studies have indicated that the occurrence of sarcopenia in patients with arthritis is significantly associated with advanced age [44]. In the present study, age was also confirmed as a significant driving factor for sarcopenia in arthritis patients, which aligns with the phenomenon of accelerated muscle loss after the age of 50 [40].

In patients with rheumatoid arthritis, elevated levels of inflammatory cytokines such as TNF-α and IL-6 may lead to alterations in the lipid metabolism profile. The SHAP analysis in this study indicated that the levels of HDL and total cholesterol are significant predictors of sarcopenia in arthritis patients. The phospholipid composition of HDL particles is altered in arthritis patients, characterized by decreased omega-3 fatty acid content and increased sphingomyelin. This change in HDL composition may enhance its stability and delay its clearance, resulting in elevated plasma concentrations but impaired function. Such dysfunctional HDL may indirectly suppress muscle protein synthesis by exacerbating the inflammatory response, potentially through a reduction in its antioxidative stress capacity [45]. Furthermore, the deposition of cholesterol and oxidized LDL (ox-LDL) in muscle tissue can induce mitochondrial dysfunction and insulin resistance, both of which are key drivers of sarcopenia in arthritis patients [46].

Previous studies have consistently reported that arthritis patients with comorbid sarcopenia exhibit a significantly lower body mass index (BMI) [3,4,5,47]. The findings of the present study support this view and confirm that a lower BMI is a strong predictor of sarcopenia, serving as a key clinical identifier. In arthritis patients, persistent chronic inflammation can impair the function of muscle satellite cells, subsequently leading to a loss of body cell mass [48]. A reduction in body cell mass is closely associated with malnutrition and decreased muscle mass. Consequently, low BMI can be regarded as both a significant outcome and a clinical manifestation of the aforementioned pathological process, where chronic inflammation contributes to muscle wasting via satellite cell dysfunction.

This study confirms that longer disease duration is a significant risk factor for sarcopenia in arthritis patients, a finding that is consistent with the results reported by Laura et al. [49]. Their study indicated that each additional year of arthritis is associated with a 10% increase in the risk of sarcopenia. Prolonged disease duration may exacerbate muscle loss through several pathways: on the one hand, chronic disease burden often leads to malnutrition, directly accelerating the reduction in muscle mass; on the other hand, persistent joint pathology leads to pain and functional limitations, resulting in a substantial decrease in physical activity. This can trap patients in a vicious cycle of ‘reduced activity → muscle atrophy → further functional decline’.

Previous studies by Tada [4] and Torii [5] have consistently demonstrated significant muscle atrophy in older adults with arthritis. This comorbid condition of arthritis and sarcopenia is closely associated with declines in physical function, particularly reduced gait speed. In alignment with these earlier findings, our study identified walking speed as a core feature in the decision tree model for assessing sarcopenia risk in arthritis patients (Figure 6A,B). As a chronic inflammatory condition, arthritis is characterized by elevated levels of proinflammatory cytokines in the systemic circulation. Among these factors, TNF-α promotes muscle protein degradation in arthritis patients through the NF-κB signaling pathway, thereby contributing to muscle loss [50]. Furthermore, long-term systemic glucocorticoid use in arthritis management represents another major contributor to sarcopenia development. The decline in muscle strength and endurance resulting from sarcopenia consequently leads to reduced walking speed during daily activities in arthritis patients, creating a bidirectional relationship between mobility impairment and disease progression.

According to a study by Hu et al. [10] utilizing CHARLS data, a significant association was observed between sarcopenia and a greater risk of cognitive impairment. Consistent with the findings of Hu et al. [10], the recall summary score was negatively correlated with sarcopenia risk in patients with arthritis. Arthritis patients often experience chronic pain, and long-term pain can affect neural plasticity and neurotransmitter balance, leading to cognitive impairment. When cognitive impairment arises, patients’ joint self-care abilities decline, and persistent joint dysfunction increases the risk of sarcopenia [51].

Building upon the work of Tu et al. [52], who established a significant longitudinal association between loneliness and a 27% higher risk of sarcopenia in a Chinese national cohort, our results extend this relationship to the arthritis population. We found that loneliness is a significant and independent predictor of the development of sarcopenia in arthritis patients, suggesting that psychosocial factors such as loneliness may exacerbate the risk of muscle loss, possibly through mechanisms such as chronic inflammation.

Using the UK Biobank cohort, Cook et al. [53] explored whether the interaction between arthritis and comorbidities increases the risk of sarcopenia. These findings indicate that the coexistence of arthritis and chronic conditions, including stroke, is associated with an increased risk of sarcogenesis. This aligns with the results of the present study, where SHAP global analysis identified the comorbidity of arthritis and stroke as a significant risk factor for sarcopenia (Figure 6A,B). The inflammatory response inherent to arthritis can stimulate immune cells to produce reactive oxygen species (ROS), thereby triggering oxidative stress. This vicious cycle of inflammation and oxidative stress may lead to multisystem dysfunction, contributing to physical frailty, decreased exercise tolerance, and ultimately, the onset of sarcopenia [35,54]. Consequently, the comorbid state of arthritis and stroke can initiate a cascade of events culminating in sarcopenia.

### 4.6. Limitations

This study has several limitations. First, the research was based on the ELSA and SHARE databases and did not employ the gold standard DXA (dual-energy X-ray absorptiometry) for measuring skeletal muscle mass. Instead, sarcopenia was diagnosed via the skeletal muscle mass index (SMI) calculation method proposed by Lee anthropometric equation [30] combined with grip strength assessment. Although this method has demonstrated good agreement with the DXA gold standard, future studies are recommended to incorporate DXA data for sarcopenia diagnosis in large-scale cohorts. It is important to interpret the proportion of sarcopenia cases in this study within the context of the longitudinal study design. Unlike cross-sectional studies which often report high prevalence rates of sarcopenia in arthritis populations (approximately 33% [55]), our study specifically excluded prevalent cases at baseline to predict the risk of incident (new-onset) sarcopenia. Consequently, the observed event rate reflects the transition from a non-sarcopenic to a sarcopenic state over the 12-year follow-up. Our observed cumulative incidence of 12.9% (253/1959) is highly consistent with other longitudinal analyses of the ELSA cohort. For instance, Ragusa et al. [28] reported a 10-year incidence of 12.1%, and other multimorbidity studies utilizing ELSA data have reported a 12-year incidence of 13.7% [56]. This consistency suggests that our study population is representative and that the identified cases accurately reflect the natural history of sarcopenia onset in older European adults.

Second, the definition of arthritis in the ELSA and SHARE cohorts relied on self-reported physician diagnosis, which prevents the differentiation between osteoarthritis, rheumatoid arthritis, and other subtypes. While this limitation is inherent to large-scale longitudinal surveys and has been accepted in recent clinical studies focusing on risk prediction in arthritis populations [25,26], we acknowledge that the underlying mechanisms of sarcopenia may differ between inflammatory and degenerative joint diseases. Therefore, our model should be viewed as a broad-spectrum screening tool for the ‘arthritis phenotype’ in primary care, rather than a subtype-specific diagnostic instrument. Future studies incorporating radiographic or serological data are warranted to refine risk prediction for specific arthritis subtypes. Third, the ELSA database is updated biennially, which can lead to infrequent updates for some core variables related to sarcopenia. Fourth, Regarding the exclusion criteria, participants with missing data exceeding 25% were removed to ensure the stability of the imputation models. However, we acknowledge that this decision may influence the sample composition. In geriatric cohort studies, missing data are rarely missing completely at random; for instance, participants unable to complete physical performance assessments (such as gait speed or grip strength) frequently exhibit greater disease severity or poorer functional status. Consequently, the exclusion of these individuals may introduce selection bias, potentially skewing the final analytical sample towards a relatively ‘healthier’ sub-population of arthritis patients. Therefore, caution should be exercised when applying this model to patients with severe functional impairment who may not align with the characteristics of the training cohort, and risk estimates in such clinical scenarios should be interpreted with care. Fifth, regarding external validation, although the SHARE cohort is multinational and includes major European nations (e.g., Germany, France, Italy), it exhibits an uneven geographical distribution with an underrepresentation of Eastern European nations. Sarcopenia prevalence and progression are known to be influenced by regional lifestyle factors (e.g., dietary patterns), socioeconomic status, and healthcare accessibility, which vary significantly across Europe. This geographical imbalance implies that the model’s generalizability to specific underrepresented regions remains to be fully verified, and further validation in more diverse European populations is warranted. Finally, the ELSA database did not include genetic data, metabolic data, or environmental exposure data. The inclusion of these variables in the future could further refine the model and enhance its predictive accuracy for sarcopenia in arthritis patients.

### 4.7. Implications for Clinical Practice

On the basis of the decision tree machine learning algorithm, this study developed a risk prediction model for sarcopenia among older European adults with arthritis. This model provides a practical and rapid screening tool for the early and precise identification of individuals with arthritis at risk of sarcopenia in community settings. After the decision tree algorithm is integrated into the electronic health record (EHR) system, healthcare providers need to input only nine simple clinical indicators, namely, stroke, BMI, HDL, loneliness, walking speed, disease duration, age, recall summary score, and total cholesterol level, to generate an individualized probability of sarcopenia risk in arthritis patients, thereby enabling risk-stratified management.

In the real-world context of primary care, the vast majority of older adults presenting to General Practitioners (GPs) with joint pain lack a definitive subtype diagnosis at the initial stage, often receiving a provisional label of ‘joint pain’ or ‘Undifferentiated Arthritis (UA)’. Incorporating complex immunological markers or confirmatory imaging findings as input variables would severely restrict the model’s accessibility and feasibility in primary care settings. Specifically, requiring diagnostic results that are typically accessible only through specialists (e.g., anti-CCP antibodies or MRI) at the screening phase would preclude the model’s implementation in community health centers, thereby undermining its clinical utility as a primary screening tool.

The 2016 update of the EULAR recommendations for the management of early arthritis [57] explicitly states: ‘Patients presenting with arthritis (any joint swelling) should be referred to, and seen by, a rheumatologist within 6 weeks after the onset of symptoms.’ Our model is designed specifically to bridge this clinical gap during the 6-week referral waiting period. By utilizing data on the ‘arthritis clinical phenotype’ (including self-reported diagnosis) that is readily available to GPs without the need for additional costly testing, the model identifies individuals at high risk of sarcopenia before a definitive diagnosis is established by a rheumatology specialist. This capability enables GPs to intervene during the waiting window for specialist appointments. Moreover, this early identification facilitates the timely prioritization of further examination of biological indicators upon specialist review [58].

Consequently, once the model generates a ‘High Risk (exceeds the cutoff value of 0.88 in Table 2)’ alert based on easily obtainable clinical indicators, such as age, walking speed, and BMI, it can guide GPs in executing precise clinical decisions: (1) Triage Referral: GPs can flag ‘high sarcopenia risk’ in referral letters to assist specialists in prioritizing patients requiring urgent intervention; (2) Initiate Prehabilitation: GPs can immediately recommend resistance exercise and nutritional support without waiting for a definitive diagnosis. This strategy is fully consistent with the European Working Group on Sarcopenia in Older People (EWGSOP2) [59] recommendations regarding early case finding, preventing patients from missing the optimal window for lifestyle intervention during the prolonged wait for a confirmed diagnosis.

## 5. Conclusions

This study developed an interpretable machine learning model for predicting sarcopenia risk in older European adults with arthritis by systematically integrating multidimensional core variables, including demographic characteristics (age), functional impairment (walking speed), comorbidities (stroke), psychosocial factors (loneliness), disease features (disease duration), cognitive function (recall summary score), and metabolic indicators (BMI, HDL, total cholesterol level). The model, built on a decision tree algorithm, demonstrated excellent performance across both the ELSA and SHARE cohorts. Its lightweight design facilitates deployment as an online tool, enabling real-time sarcopenia risk assessment in both community and specialized clinical settings.

## Figures and Tables

**Table 1:** Baseline characteristics of the training and internal validation sets.

Variable	Training Set	Internal Validation Set	*p* ^1^	SMDs ^2^
N	1371	588	0.714	0.023
Sarcopenia (%)	177 (12.91)	76 (12.93)	0.993	<0.001
Age (median [IQR])	71.00 [68.00, 77.00]	72.00 [68.00, 78.00]	0.055	0.084
Gender, Female (%)	687 (50.1)	291 (49.5)	0.802	0.012
Stroke, Yes (%)	72 (5.3)	34 (5.8)		
BMI (mean (SD))	28.14 (4.88)	28.19 (4.99)	0.79	0.013
HDL (mean (SD))	1.58 (0.59)	1.57 (0.56)	0.903	0.008
Loneliness (mean (SD))	1.37 (0.39)	1.39 (0.39)	0.326	0.054
Walking_speed_test (mean (SD))	3.17 (1.75)	3.25 (1.97)	0.404	0.045
Disease_duration (median [IQR])	16.000 [11.000, 25.000]	17.000 [9.000, 23.000]	0.432	0.078
Recall_summary_score (mean (SD))	10.13 (3.58)	10.07 (3.59)	0.627	0.024
Total_cholesterol_level (mean (SD))	5.01 (1.39)	4.97 (1.29)	0.686	0.026

Note: Values are presented as mean (standard deviation) for continuous variables with normal distribution, median [interquartile range] for skewed variables, and number (percentage) for categorical variables. ^1^ *p*-values were calculated using the *t*-test or Mann–Whitney U test for continuous variables and the Chi-square test for categorical variables. ^2^ SMD, Standardized Mean Difference. An SMD < 0.1 indicates excellent balance between groups.

**Table 2:** Evaluation of the performance of the six algorithms in the training set and validation set.

Category	AUC (95%CI)	Cutoff (95%CI)	Precision (95%CI)	Sensitivity (95%CI)	Specificity (95%CI)	PPV (95%CI)	NPV (95%CI)	F1 Score (95%CI)
Training set								
XGBoost	0.956 (0.937–0.974)	0.629 (0.615–0.642)	0.96 (0.954–0.966)	0.969 (0.962–0.977)	0.901 (0.895–0.906)	0.985 (0.984–0.986)	0.816 (0.781–0.851)	0.977 (0.973–0.981)
logistic	0.887 (0.867–0.906)	0.781 (0.777–0.785)	0.896 (0.894–0.899)	0.904 (0.900–0.907)	0.847 (0.842–0.851)	0.975 (0.975–0.976)	0.567 (0.558–0.575)	0.938 (0.937–0.940)
LightGBM	0.798 (0.754–0.843)	0.969 (0.969–0.970)	0.961 (0.961–0.962)	0.999 (0.999–1.000)	0.704 (0.699–0.709)	0.958 (0.957–0.959)	0.993 (0.991–0.995)	0.978 (0.978–0.979)
RandomForest	1.000 (1.000–1.000)	0.681 (0.647–0.715)	0.998 (0.998–0.999)	0.998 (0.998–0.998)	1.0 (1.000–1.000)	1.0 (1.000–1.000)	0.988 (0.987–0.990)	0.999 (0.999–0.999)
DecisionTree	0.991 (0.987–0.995)	0.88 (0.872–0.887)	0.945 (0.939–0.952)	0.947 (0.938–0.956)	0.934 (0.921–0.947)	0.99 (0.988–0.992)	0.727 (0.698–0.757)	0.968 (0.964–0.972)
KNN	1.000 (1.000–1.000)	1.0 (1.000–1.000)	0.999 (0.998–0.999)	0.998 (0.998–0.999)	1.0 (1.000–1.000)	1.0 (1.000–1.000)	0.989 (0.987–0.990)	0.999 (0.999–0.999)
Validation set								
XGBoost	0.933 (0.871–0.992)	0.629 (0.615–0.642)	0.936 (0.929–0.944)	0.951 (0.942–0.961)	0.834 (0.789–0.879)	0.975 (0.968–0.982)	0.723 (0.687–0.760)	0.963 (0.958–0.967)
logistic	0.882 (0.822–0.943)	0.781 (0.777–0.785)	0.891 (0.886–0.897)	0.901 (0.894–0.908)	0.826 (0.800–0.852)	0.972 (0.968–0.976)	0.554 (0.535–0.572)	0.935 (0.932–0.939)
LightGBM	0.796 (0.665–0.928)	0.969 (0.969–0.970)	0.96 (0.954–0.966)	0.999 (0.997–1.000)	0.7 (0.648–0.752)	0.957 (0.950–0.964)	0.99 (0.977–1.003)	0.978 (0.974–0.981)
RandomForest	0.953 (0.899–0.999)	0.681 (0.647–0.715)	0.966 (0.958–0.974)	0.987 (0.979–0.996)	0.822 (0.772–0.872)	0.974 (0.967–0.981)	0.913 (0.861–0.965)	0.98 (0.976–0.985)
DecisionTree	0.921 (0.848–0.988)	0.88 (0.872–0.887)	0.916 (0.893–0.940)	0.928 (0.906–0.950)	0.838 (0.789–0.888)	0.975 (0.967–0.983)	0.646 (0.578–0.715)	0.95 (0.936–0.965)
KNN	0.929 (0.860–0.993)	1.0 (1.000–1.000)	0.853 (0.842–0.864)	0.848 (0.833–0.863)	0.885 (0.848–0.923)	0.981 (0.974–0.987)	0.466 (0.447–0.486)	0.909 (0.902–0.917)

Note: The 95% Confidence Intervals (CIs) for all performance metrics were calculated using bootstrapping with 1000 resamples to ensure robust statistical estimation. Caution regarding overfitting: The perfect performance metrics (AUC = 1.000) observed for Random Forest and KNN in the training set indicate severe overfitting. These values are reported for completeness but were not used for model selection. The optimal model was selected solely on the basis of its performance in the internal validation set to ensure generalizability. AUC: Area Under the Curve; Positive Predictive Value, PPV; Negative Predictive Value, NPV.

## Data Availability

The original contributions presented in this study are included in the article/Supplementary Materials. Further inquiries can be directed to the author.

## Supplementary Materials

The following supporting information can be downloaded at: https://www.mdpi.com/article/10.3390/jcm15031022/s1, Figure S1: Flowchart of participant inclusion and exclusion for the ELSA dataset; Figure S2: Flowchart of participant inclusion and exclusion for the SHARE dataset; Figure S3: Schematic illustration of the prospective longitudinal study design and temporal architecture; Figure S4: External validation of the DecisionTree classification from the SHARE dataset. (A) ROC Curve for the external validation set; (B) Calibration Plot; (C) Test Decision Curve Analysis. Table S1: TRIPOD Checklist: Prediction Model Development and Validation; Table S2: Random-forest imputation; Table S3: Baseline characteristics in training set and internal validation set; Table S4: Selection frequency across cross-validation folds; Table S5: Parameter Selection for Machine-Learning Models; Table S6: Baseline characteristics in training set, internal validation set and external validation set.
