# Supplementary material for: Machine Learning-Driven Personalized Risk Prediction: Developing an Explainable Sarcopenia Model for Older European Adults with Arthritis
Source: J Clin Med. 2026 Jan 27;15(3):1022. doi: 10.3390/jcm15031022 (PMC12897810; doi:10.3390/jcm15031022)
Supplement: Supplementary file 1 [file jcm-15-01022-s001.zip › Supplementary_tables/Supplementary_File_TableS3_Baseline_Characteristic2.pdf]

Supplementary Table S3. Baseline characteristics in training set and internal validation set.

Variables	**Category**	**Overall (n=1959)**	**Training set (n=1371)**	**Validation set (n=588)**	**Statistic**	***P* value**
Gender ,n(%)	Male	981(50.077)	684(49.891)	297(50.510)	0.063	0.802
	Female	978(49.923)	687(50.109)	291(49.490)		
Marital_status ,n(%)	Have a spouse	1670(93.244)	1170(93.525)	500(92.593)	0.521	0.471
	No spouse	121(6.756)	81(6.475)	40(7.407)		
ADL ,n(%)	0.0	1460(83.812)	1019(83.593)	441(84.321)	4.691	0.584
	1.0	151(8.668)	108(8.860)	43(8.222)		
	2.0	51(2.928)	39(3.199)	12(2.294)		
	3.0	27(1.550)	20(1.641)	7(1.338)		
	4.0	23(1.320)	16(1.313)	7(1.338)		
	5.0	16(0.918)	8(0.656)	8(1.530)		
	6.0	14(0.804)	9(0.738)	5(0.956)		
IADL ,n(%)	0.0	1435(80.078)	1010(80.735)	425(78.558)	3.969	0.860
	1.0	169(9.431)	114(9.113)	55(10.166)		
	2.0	76(4.241)	55(4.396)	21(3.882)		
	3.0	43(2.400)	27(2.158)	16(2.957)		
	4.0	16(0.893)	12(0.959)	4(0.739)		
	5.0	10(0.558)	6(0.480)	4(0.739)		
	6.0	11(0.614)	7(0.560)	4(0.739)		
	7.0	10(0.558)	7(0.560)	3(0.555)		
	8.0	22(1.228)	13(1.039)	9(1.664)		
Employment ,n(%)	Yes	1564(87.326)	1094(87.450)	470(87.037)	0.058	0.809
	No	227(12.674)	157(12.550)	70(12.963)		
Sarcopenia ,n(%)	Yes	253(12.915)	177(12.910)	76(12.925)	0.000	0.993

Variables	Category	Overall (n=1959)	Training set (n=1371)	Validation set (n=588)	Statistic	*P* value
	No	1706(87.085)	1194(87.090)	512(87.075)		
Diabetes ,n(%)	Yes	1610(90.095)	1120(89.744)	490(90.909)	0.573	0.449
	No	177(9.905)	128(10.256)	49(9.091)		
Hypertension ,n(%)	Yes	1083(61.186)	759(61.358)	324(60.788)	0.051	0.821
	No	687(38.814)	478(38.642)	209(39.212)		
Hip_fracture ,n(%)	Yes	43(2.404)	31(2.484)	12(2.218)	0.114	0.736
	No	1746(97.596)	1217(97.516)	529(97.782)		
Osteoporosis ,n(%)	Yes	1681(95.187)	1171(95.203)	510(95.149)	0.002	0.961
	No	85(4.813)	59(4.797)	26(4.851)		
Fallen_down ,n(%)	Yes	1263(74.120)	889(74.958)	374(72.201)	1.429	0.232
	No	441(25.880)	297(25.042)	144(27.799)		
Pain ,n(%)	No pain	966(56.624)	676(56.902)	290(55.985)	0.467	0.926
	Mild	213(12.485)	149(12.542)	64(12.355)		
	Moderate	390(22.860)	271(22.811)	119(22.973)		
	Severe	137(8.030)	92(7.744)	45(8.687)		
Education ,n(%)	Hight school	655(36.128)	449(35.161)	206(38.433)	5.439	0.142
	High school graduate	395(21.787)	294(23.023)	101(18.843)		
	Some colleges	446(24.600)	319(24.980)	127(23.694)		
	College above	317(17.485)	215(16.836)	102(19.030)		
Depression ,n(%)	Yes	252(12.864)	174(12.691)	78(13.265)	0.121	0.728
	No	1707(87.136)	1197(87.309)	510(86.735)		
Smoken ,n(%)	Yes	1676(93.999)	1167(93.886)	509(94.259)	0.093	0.760
	No	107(6.001)	76(6.114)	31(5.741)		

Variables	Category	Overall (n=1959)	Training set (n=1371)	Validation set (n=588)	Statistic	*P* value
Drink ,n(%)	Yes	1036(65.861)	716(65.150)	320(67.511)	0.821	0.365
	No	537(34.139)	383(34.850)	154(32.489)		
Heart_problems ,n(%)	Yes	1342(74.888)	938(74.980)	404(74.677)	0.018	0.892
	No	450(25.112)	313(25.020)	137(25.323)		
Stroke ,n(%)	Yes	106(5.915)	72(5.755)	34(6.285)	0.190	0.663
	No	1686(94.085)	1179(94.245)	507(93.715)		
Lung_disease ,n(%)	Yes	1647(91.908)	1152(92.086)	495(91.497)	0.176	0.675
	No	145(8.092)	99(7.914)	46(8.503)		
Dementia ,n(%)	Yes	1770(98.772)	1238(98.961)	532(98.336)	1.214	0.270
	No	22(1.228)	13(1.039)	9(1.664)		
Age ,median[IQR]		72.000[68.000,77.000]	71.000[68.000,77.000]	72.000[68.000,78.000]	-1.917	0.055
Disease_duration ,median[IQR]		17.000[10.000,24.000]	16.000[11.000,25.000]	17.000[9.000,23.000]	0.786	0.432
Total_cholesterol_level (mmol/l) ,median[IQR]		5.000[4.300,5.800]	5.100[4.300,5.800]	4.900[4.200,5.800]	0.689	0.491
HDL (mmol/l) , (mean (SD))		1.58 (0.59)	1.58 (0.59)	1.57 (0.56)	0.452	0.903
Triglyceride level (mmol/l), median[IQR]		1.300[1.000,1.800]	1.300[0.900,1.800]	1.300[1.000,1.800]	-0.495	0.620
CRP (mg/l), median[IQR]		1.300[0.700,3.000]	1.300[0.700,3.000]	1.300[0.600,3.000]	0.429	0.668
Haemoglobin_level (g/dl) ,median[IQR]		140.000[131.000,149.000]	140.000[131.000,149.000]	139.000[131.000,148.000]	0.343	0.731
HbA1C (%), median[IQR]		39.000[36.000,42.000]	39.000[36.000,42.000]	38.000[36.000,41.000]	0.524	0.599
BMI , (mean (SD))		28.14 (4.88)	28.12 (4.83)	28.19 (4.99)	-0.144	0.79
Walking_speed_test (m/s), median (mean (SD))		3.20 (1.82)	3.17 (1.75)	3.25 (1.97)	0.072	0.404
Loneliness, (mean (SD))		1.380 (0.39)	1.37 (0.39)	1.39 (0.39)	-0.992	0.326
Recall_summary_score ,median[IQR]		10.13(3.58)	10.13(3.58)	10.07(3.59)	0.764	0.627
Verbal_fluency_score ,median[IQR]		5.00 (1.36)	5.01 (1.39)	4.97 (1.29)	0.238	0.686

Note: ADL: Activities of Daily Living; IADL: Instrumental Activities of Daily Living; IQR: Interquartile Range
