# Supplementary material for: Machine Learning-Driven Personalized Risk Prediction: Developing an Explainable Sarcopenia Model for Older European Adults with Arthritis
Source: J Clin Med. 2026 Jan 27;15(3):1022. doi: 10.3390/jcm15031022 (PMC12897810; doi:10.3390/jcm15031022)
Supplement: Supplementary file 1 [file jcm-15-01022-s001.zip › Supplementary_tables/Supplementary_File_TableS5_ML_parameter.pdf]

Supplementary File TableS5
Parameter Selection for Machine-Learning Models

1. XGBClassifier:

colsample_bytree: 1
learning_rate: 0.1
max_depth: 8
min_child_weight: 2
n_estimators: 5
reg_lambda: 1
subsample: 1

2. LogisticRegression:

C: 0.01
l1_ratio: None
max_iter: 50
penalty: l2
solver: lbfgs
tol: 0.0001

3. LGBMClassifier

boosting_type: gbdt
learning_rate: 2
max_depth: 1
n_estimators: 5
num_leaves: 5

4. RandomForestClassifier

criterion: gini
max_depth: None
max_features: sqrt
min_impurity_decrease: 0.0
min_samples_leaf: 1
min_samples_split: 2
n_estimators: 100

5. KNeighborsClassifier

algorithm: auto
leaf_size: 10
n_neighbors: 6
p: 2
weights: uniform

6. DecisionTreeClassifier

criterion: gini

max_depth: 20

min_samples_leaf: 1

min_samples_split: 50
