# Supplementary material for: Machine Learning-Driven Personalized Risk Prediction: Developing an Explainable Sarcopenia Model for Older European Adults with Arthritis
Source: J Clin Med. 2026 Jan 27;15(3):1022. doi: 10.3390/jcm15031022 (PMC12897810; doi:10.3390/jcm15031022)
Supplement: Supplementary file 1 [file jcm-15-01022-s001.zip › Supplementary_tables/Supplementary_File_TableS6_Baseline characteristics in training set, internal validation set and external validation set.pdf]

Supplementary Table S6 Baseline characteristics in training set, internal validation set and external validation set

**Variables**	**Category**	**ELSA (Training) (n=1,371)**	**ELSA (Internal Validation) (n=588)**	**SHARE (External Validation) (n=1,001)**
Gender ,n(%)	Male	684(49.891)	297(50.510)	459(45.854)
	Female	687(50.109)	291(49.490)	542(54.146)
Marital_status ,n(%)	Have a spouse	1170(93.525)	500(92.593)	723(72.23)
	No spouse	81(6.475)	40(7.407)	278(27.77)
ADL ,n(%)	0.0	1019(83.593)	441(84.321)	865(86.414)
	1.0	108(8.860)	43(8.222)	69(6.893)
	2.0	39(3.199)	12(2.294)	28(2.797)
	3.0	20(1.641)	7(1.338)	16(1.598)
	4.0	16(1.313)	7(1.338)	10(0.999)
	5.0	8(0.656)	8(1.530)	1(0.100)
	6.0	9(0.738)	5(0.956)	12 (1.198)
IADL ,n(%)	0.0	1010(80.735)	425(78.558)	842(84.116)
	1.0	114(9.113)	55(10.166)	66(6.593)
	2.0	55(4.396)	21(3.882)	32(3.197)
	3.0	27(2.158)	16(2.957)	17(1.698)
	4.0	12(0.959)	4(0.739)	6(0.599)
	5.0	6(0.480)	4(0.739)	6(0.599)
	6.0	7(0.560)	4(0.739)	12(1.199)
	7.0	7(0.560)	3(0.555)	10(0.999)
	8.0	13(1.039)	9(1.664)	10(0.999)
Employment ,n(%)	Yes	1094(87.450)	470(87.037)	764(77.094)
	No	157(12.550)	70(12.963)	227(22.906)
Sarcopenia ,n(%)	Yes	177(12.910)	76(12.925)	151(15.085)
	No	1194(87.090)	512(87.075)	850(84.915)
Diabetes ,n(%)	Yes	1120(89.744)	490(90.909)	870(86.913)
	No	128(10.256)	49(9.091)	131(13.087)
Hypertension ,n(%)	Yes	759(61.358)	324(60.788)	552(55.145)

	No	478(38.642)	209(39.212)	449(44.855)
Hip_fracture ,n(%)	Yes	31(2.484)	12(2.218)	39(3.908)
	No	1217(97.516)	529(97.782)	959(96.092)
Fallen_down ,n(%)	Yes	889(74.958)	374(72.201)	53(5.295)
	No	297(25.042)	144(27.799)	948(94.705)
Pain ,n(%)	No pain	676(56.902)	290(55.985)	779(78.056)
	Mild	149(12.542)	64(12.355)	70(7.014)
	Moderate	271(22.811)	119(22.973)	108(10.822)
	Severe	92(7.744)	45(8.687)	41(4.108)
Education ,n(%)	Hight school	449(35.161)	206(38.433)	310(30.989)
	High school graduate	294(23.023)	101(18.843)	208(20.789)
	Some colleges	319(24.980)	127(23.694)	277(27.682)
	College above	215(16.836)	102(19.030)	206(20.540)
**Depression ,n(%)**	Yes	174(12.691)	78(13.265)	157(15.684)
	No	1197(87.309)	510(86.735)	844(84.316)
Smoken ,n(%)	Yes	1167(93.886)	509(94.259)	481(48.052)
	No	76(6.114)	31(5.741)	520(51.948)
Drink ,n(%)	Yes	716(65.150)	320(67.511)	407(40.659)
	No	383(34.850)	154(32.489)	594(59.341)
Heart_problems ,n(%)	Yes	938(74.980)	404(74.677)	827(82.617)
	No	313(25.020)	137(25.323)	174(17.383)
Stroke ,n(%)	Yes	1179(94.245)	507(93.715)	933(93.207)
	No	72(5.755)	34(6.285)	68(6.793)
Lung_disease ,n(%)	Yes	1152(92.086)	495(91.497)	933(93.207)
	No	99(7.914)	46(8.503)	68(6.793)
Dementia ,n(%)	Yes	1238(98.961)	532(98.336)	979(97.802)
	No	13(1.039)	9(1.664)	22(2.198)
Age ,median[IQR]		71.000[68.000,77.000]	72.000[68.000,78.000]	72.000[68.000,79.000]
Total_cholesterol_level (mmol/l) ,median[IQR]		5.100[4.300,5.800]	4.900[4.200,5.800]	5.756[5.328,6.187]
HDL (mmol/l) , median[IQR]		1.500[1.300,1.900]	1.500[1.200,1.900]	1.679[1.550,1.875]
Triglyceride level (mmol/l), median[IQR]		1.300[0.900,1.800]	1.300[1.000,1.800]	1.300[0.900,1.800]

CRP (mg/l), median[IQR]		1.300[0.700,3.000]	1.300[0.600,3.000]	1.941[1.446,3.025]
BMI ,median[IQR]		27.380[24.582,30.685]	27.463[24.250,30.695]	25.236[23.389,27.755]
Walking_speed_test (m/s), median[IQR]		2.720[2.340,3.390]	2.695[2.300,3.380]	0.757±0.278
Loneliness ,median[IQR]		1.250[1.000,1.500]	1.250[1.000,1.500]	3.000[3.000,4.000]
Recall_summary_score ,median[IQR]		11.000[8.000,13.000]	11.000[8.000,13.000]	10.000[7.000,12.000]
Verbal_fluency_score ,median[IQR]		21.000[17.000,26.000]	21.000[17.000,26.000]	19.000[14.000,25.000]

Note: ADL: Activities of Daily Living; IADL: Instrumental Activities of Daily Living; IQR: Interquartile Range

**Variables**	**Category**	**ELSA (Training) (n=1,371)**	**ELSA (Internal Validation) (n=588)**	**SHARE (External Validation) (n=1,001)**
Gender ,n(%)	Male	684(49.891)	297(50.510)	459(45.854)
	Female	687(50.109)	291(49.490)	542(54.146)
Marital_status ,n(%)	Have a spouse	1170(93.525)	500(92.593)	723(72.23)
	No spouse	81(6.475)	40(7.407)	278(27.77)
ADL ,n(%)	0.0	1019(83.593)	441(84.321)	865(86.414)
	1.0	108(8.860)	43(8.222)	69(6.893)
	2.0	39(3.199)	12(2.294)	28(2.797)
	3.0	20(1.641)	7(1.338)	16(1.598)
	4.0	16(1.313)	7(1.338)	10(0.999)
	5.0	8(0.656)	8(1.530)	1(0.100)
	6.0	9(0.738)	5(0.956)	12 (1.198)
IADL ,n(%)	0.0	1010(80.735)	425(78.558)	842(84.116)
	1.0	114(9.113)	55(10.166)	66(6.593)
	2.0	55(4.396)	21(3.882)	32(3.197)
	3.0	27(2.158)	16(2.957)	17(1.698)
	4.0	12(0.959)	4(0.739)	6(0.599)

Variables	Category	ELSA (Training) (n=1,371)	ELSA (Internal Validation) (n=588)	SHARE (External Validation) (n=1,001)
	5.0	6(0.480)	4(0.739)	6(0.599)
	6.0	7(0.560)	4(0.739)	12(1.199)
	7.0	7(0.560)	3(0.555)	10(0.999)
	8.0	13(1.039)	9(1.664)	10(0.999)
Employment ,n(%)	Yes	1094(87.450)	470(87.037)	764(77.094)
	No	157(12.550)	70(12.963)	227(22.906)
Sarcopenia ,n(%)	Yes	177(12.910)	76(12.925)	151(15.085)
	No	1194(87.090)	512(87.075)	850(84.915)
Diabetes ,n(%)	Yes	1120(89.744)	490(90.909)	870(86.913)
	No	128(10.256)	49(9.091)	131(13.087)
Hypertension ,n(%)	Yes	759(61.358)	324(60.788)	552(55.145)
	No	478(38.642)	209(39.212)	449(44.855)
Hip_fracture ,n(%)	Yes	31(2.484)	12(2.218)	39(3.908)

**Variables**	**Category**	**ELSA (Training) (n=1,371)**	**ELSA (Internal Validation) (n=588)**	**SHARE (External Validation) (n=1,001)**
	No	1217(97.516)	529(97.782)	959(96.092)
Fallen_down ,n(%)	Yes	889(74.958)	374(72.201)	53(5.295)
	No	297(25.042)	144(27.799)	948(94.705)
Pain ,n(%)	No pain	676(56.902)	290(55.985)	779(78.056)
	Mild	149(12.542)	64(12.355)	70(7.014)
	Moderate	271(22.811)	119(22.973)	108(10.822)
	Severe	92(7.744)	45(8.687)	41(4.108)
Education ,n(%)	Hight school	449(35.161)	206(38.433)	310(30.989)
	High school graduate	294(23.023)	101(18.843)	208(20.789)
	Some colleges	319(24.980)	127(23.694)	277(27.682)
	College above	215(16.836)	102(19.030)	206(20.540)
**Depression ,n(%)**	Yes	174(12.691)	78(13.265)	157(15.684)
	No	1197(87.309)	510(86.735)	844(84.316)

Variables	Category	ELSA (Training) (n=1,371)	ELSA (Internal Validation) (n=588)	SHARE (External Validation) (n=1,001)
Smoken ,n(%)	Yes	1167(93.886)	509(94.259)	481(48.052)
	No	76(6.114)	31(5.741)	520(51.948)
Drink ,n(%)	Yes	716(65.150)	320(67.511)	407(40.659)
	No	383(34.850)	154(32.489)	594(59.341)
Heart_problems ,n(%)	Yes	938(74.980)	404(74.677)	827(82.617)
	No	313(25.020)	137(25.323)	174(17.383)
Stroke ,n(%)	Yes	1179(94.245)	507(93.715)	933(93.207)
	No	72(5.755)	34(6.285)	68(6.793)
Lung_disease ,n(%)	Yes	1152(92.086)	495(91.497)	933(93.207)
	No	99(7.914)	46(8.503)	68(6.793)
Dementia ,n(%)	Yes	1238(98.961)	532(98.336)	979(97.802)
	No	13(1.039)	9(1.664)	22(2.198)
Age ,median[IQR]		71.000[68.000,77.000]	72.000[68.000,78.000]	72.000[68.000,79.000]

Variables	Category	ELSA (Training) (n=1,371)	ELSA (Internal Validation) (n=588)	SHARE (External Validation) (n=1,001)
Total_cholesterol_level (mmol/l) ,median[IQR]		5.100[4.300,5.800]	4.900[4.200,5.800]	5.756[5.328,6.187]
HDL (mmol/l) , median[IQR]		1.500[1.300,1.900]	1.500[1.200,1.900]	1.679[1.550,1.875]
Triglyceride level (mmol/l), median[IQR]		1.300[0.900,1.800]	1.300[1.000,1.800]	1.300[0.900,1.800]
CRP (mg/l), median[IQR]		1.300[0.700,3.000]	1.300[0.600,3.000]	1.941[1.446,3.025]
BMI ,median[IQR]		27.380[24.582,30.685]	27.463[24.250,30.695]	25.236[23.389,27.755]
Walking_speed_test (m/s), median[IQR]		2.720[2.340,3.390]	2.695[2.300,3.380]	0.757±0.278
Loneliness ,median[IQR]		1.250[1.000,1.500]	1.250[1.000,1.500]	3.000[3.000,4.000]
Recall_summary_score ,median[IQR]		11.000[8.000,13.000]	11.000[8.000,13.000]	10.000[7.000,12.000]
Verbal_fluency_score ,median[IQR]		21.000[17.000,26.000]	21.000[17.000,26.000]	19.000[14.000,25.000]
