# Supplementary material for: Machine Learning-Driven Personalized Risk Prediction: Developing an Explainable Sarcopenia Model for Older European Adults with Arthritis
Source: J Clin Med. 2026 Jan 27;15(3):1022. doi: 10.3390/jcm15031022 (PMC12897810; doi:10.3390/jcm15031022)
Supplement: Supplementary file 1 [file jcm-15-01022-s001.zip › Supplementary_Method.pdf]

2. Materials and Methods

2.1 Data Origin

The data utilized in this research stem from the English Longitudinal Study of Ageing (ELSA) and Survey of Health, Ageing and Retirement in Europe (SHARE).

English Longitudinal Study of Ageing (ELSA) is a multidisciplinary biennial panel study that follows a nationally-representative sample of community-dwelling adults aged 50 years and older (and their partners) living in private households in England. The original sample was drawn from respondents to the Health Survey for England (HSE) 1998–2001, and the cohort has been refreshed at Waves 3, 4, 6, 7, 8, 9 and 10 to maintain population representativeness. Data collection occurs every two years and includes face-to-face computer-assisted personal interviews (CAPI), self-completion questionnaires, and a nurse visit that measures physical function, anthropometry and biomarkers.

Survey of Health, Ageing and Retirement in Europe (SHARE) is the largest cross-national panel study of individuals aged 50 years and older in 28 European countries and Israel. Initiated in 2004 and fielded every two years, SHARE uses ex-ante harmonised questionnaires and interview software to ensure strict comparability across countries. The project is organised as the first European Research Infrastructure Consortium (SHARE-ERIC) and is harmonised with ELSA and the U.S. Health and Retirement Study (HRS). Data are freely available to the scientific community for research on health, economic status and social networks in ageing populations.

2.2 Study Participants

(1) Inclusion criteria: ① age $\geq$ 65 years; ② diagnosed with arthritis at baseline; ③ not diagnosed with sarcopenia at cohort baseline. (2) Exclusion criteria: ① missing data on arthritis diagnosis at baseline or during follow-up; ② missing data on components of the sarcopenia diagnostic criteria at baseline or during follow-up; ③ key variables in the variable screening with a missing value proportion exceeding 25% [1]. After applying the predefined inclusion and exclusion criteria, a total of 1959 participants from the ELSA dataset were ultimately included for machine learning modeling and internal validation, and 1001 participants from the SHARE dataset were used for external validation of the model. **Figure S1** and **Figure S2** respectively show the flowcharts of study participant inclusion and exclusion for the ELSA and SHARE databases.

2.3. Research Variables

2.3.1. Outcome Variable

Consistent with prior ELSA and SHARE analyses[2,3], body composition was not assessed using dual-energy X-ray absorptiometry (DXA). Instead, we applied the low skeletal muscle mass index (SMI) proposed by Veronese *et al.*[4], which has demonstrated strong agreement with DXA-derived measures. Sarcopenia was diagnosed when both of the following criteria were met: (1) low hand-grip strength, defined as the mean of three trials with a Smedley dynamometer performed on the dominant hand (< 27 kg for men and < 16 kg for women); and (2) low skeletal muscle mass, defined as the lowest sex-specific quartile of the skeletal muscle index (SMI). SMI was calculated as skeletal muscle mass (SMM) divided by body mass index (BMI). SMM was estimated using the Veronese *et al* equation[4]: $SMM = 0.244 \times weight + 7.8 \times height + 6.6 \times sex - 0.098 \times age + race - 3.3$, where sex is coded as 1 for male and 0 for female, and race as 0 (White/Hispanic), 1.9 (Black), or −1.6 (Asian).

2.3.2. Predictor Variables

2.3.2.1 General demographic and socioeconomic factors

Demographic characteristics considered include age, gender, marital status, education level, and employment.

2.3.2.2 Arthritis-related symptoms

(1) Physical function

① 6-item summary activities of daily living (ADL) disability score: Participants were asked about their ability to perform tasks such as walking across a room, dressing, bathing or showering, eating (e.g., cutting food), getting in or out of bed, and using the toilet. ADL disability was defined as difficulty or inability to perform these activities. A summary ADL disability score was created for each participant (range 0–6; 0 indicates no disability).

② Instrumental Activities of Daily Living (IADL): The IADL includes using a map, using the telephone, taking medications, shopping for groceries, preparing meals, doing work around the house or garden, recognising physical danger, and communicating via speech, hearing or vision. A

summary IADL disability score was created for each participant (range 0–8; 0 indicates no disability).

③ **Walking speed:** Walking speed was measured by the time taken to walk 2.4 m at a habitual pace, either with or without a gait-assistance device, on a flat surface in the participants' private homes.

(2) Hip fracture status

(3) Osteoporosis

(4) Disease duration: Survey year minus the self-reported year of first diagnosis of arthritis

(5) Pain: No pain, mild pain, moderate pain and severe pain.

(5) Comorbidity: hypertension, diabetes, heart problems, stroke and lung disease.

(6) Fall

(7) Body mass index: BMI was calculated with the following formula: weight (kg) / height^2 (m^2).

2.3.2.3. Cognitive and social factors

(1) Dementia: dementia, and normal cognition.

(2) Verbal fluency score and recall summary score: Verbal fluency was assessed by asking how many different animals the participants could indicate in 60 s. Memory, as assessed with the word recall summary score, calculated as the sum of immediate and delayed verbal memory. Each participant was presented with a list of 10 nouns on a computer, one every 2 s. Participants were asked to recall as many words as possible immediately and again after a short delay during which they carried out the other cognitive tests [5].

(3) Social lioness: loneliness is gauged with the four-item UCLA Loneliness Scale[6].

2.3.2.4. Blood measurements: Total cholesterol level, HDL, Triglyceride level, CRP, Haemoglobin level, and HbA1C.

Blood samples were collected and sent to the Biochemistry Department at the Royal Victoria Infirmary, Newcastle, UK for laboratory analysis. Total cholesterol (Cholesterol Oxidase assay method), high-density lipoprotein cholesterol (direct method), and triacylglycerol (enzymatic method) levels were measured using the Olympus 640 analyser calibrated to the center for disease control guidelines. Circulating high-sensitivity C-reactive protein (CRP) was assessed using the N latex CRP mono immunoassay on the Dade Behring Nephelometer II Analyser and conducted in line with the quality control guidelines specified in the Health Survey of England technical report. Total HbA1C was measured by the Haematology Department at the Royal Victoria Infirmary using a Tosoh G7 analyser (Tosoh, Tokyo, Japan). The analytical methods used for HbA_{1c} measurement in the UK are required to be traceable to the work carried out in the Diabetes Control and Complications Trial (DCCT), part of the National Glycohemoglobin Standardization Program in the USA.

2.3.2.5. Psychological and behavioral factors

(1) Depression: Depressive symptoms are evaluated using the 8-item Centre for Epidemiologic Studies Depression Scale (CES-D-8). Respondents answer eight yes/no items, yielding a total score ranging from 0 to 8, with a score ≥ 3 commonly taken to indicate clinically significant depressive symptoms [7].

(2) Behavioral factors such as drinking and smoking habits were assessed.

2.4. Statistical Analysis

This research utilized R Studio software (glmnet 4.1.2, rmda 1.6, ggplot2 3.5.1) and Python software (scikit-learn 1.1.3, xgboost 2.0.1, lightgbm 3.2.1, shap 0.43.0) for the data analysis. The individuals included were randomly divided into a training set and an internal validation set in a 7:3 ratio using a random number method. The dataset presented count data as both numerical values

and proportions, which were analyzed using the chi-squared test or Fisher exact test for expected frequencies below 10. Normally distributed data was expressed as mean and standard deviation, subjected to student's t-test for comparison. For non-normally distributed continuous data, median and interquartile range were used, and the Mann-Whitney U test was applied for comparison. The significance level was set at $\alpha=0.05$.

The training set underwent LASSO to select significant features from the initial 33 variables. To more robustly identify key variables, this study adopted a model consensus strategy: the variable importance rankings were evaluated separately using a random forest (RF) regressor, an extreme gradient boosting classifier (XGBoost), and a ridge regressor. The top 12 variables from each model were extracted, and the most critical core variables, consistently identified by all models, were determined by taking the intersection of their Venn diagrams.

Six machine learning algorithms, including XGBoost, Logistic, KNN, Decision Tree, LightGBM, Random Forest were chosen to develop sarcopenia risk prediction models for European elderly arthritis individuals. The optimal model was selected based on evaluation metrics such as the area under the Receiver Operating Characteristic (ROC) curve, specificity, precision, sensitivity, specificity, and F1 score in the validation set [8].

Model optimization was done using 10-fold cross-validation in the training set and validated using a testing set comprising 15% of the dataset. Learning curves were utilized to assess the model's fit and stability in the training and validation sets[9]. Decision curve analysis (DCA) plots were created using R software (rmda 1.6) to aid in model selection based on clinical applicability[10]. Precision-recall (PR) curves were generated in Python to evaluate model performance, with the area under the PR curve providing additional insights into model evaluation[11]. SHAP interpretation was used in Python to analyze the importance and contribution of features to the model's predictions [12]. The external dataset was evaluated based on evaluation metrics such as the area under the Receiver Operating Characteristic (ROC) curve, specificity, precision, sensitivity, specificity, and F1 score.

Furthermore, an online risk calculator was developed based on the best-performing model to predict sarcopenia risk in middle-aged and elderly arthritis patients using newly entered patient data, thereby facilitating access for clinicians to assess sarcopenia risk in this patient population.

References

1. Alcazer V, Le Meur G, Roccon M, et al. Evaluation of a machine-learning model based on laboratory parameters for the prediction of acute leukaemia subtypes: a multicentre model development and validation study in France. *Lancet Digit Health.* 2024;6(5):e323-e333. doi: 310.1016/S2589-7500(1024)00044-X.
2. Ragusa FS, Veronese N, Vernuccio L, et al. Mild cognitive impairment predicts the onset of Sarcopenia: a longitudinal analysis from the English Longitudinal Study on Ageing. *Aging Clin Exp Res.* 2024;36(1):129. doi: 110.1007/s40520-40024-02781-z.
3. Pavón-Pulido N, Dominguez L, Blasco-García JD, et al. Identification of Predictors of Sarcopenia in Older Adults Using Machine Learning: English Longitudinal Study of Ageing. *J Clin Med.* 2024;13(22):6794. doi: 6710.3390/jcm13226794.
4. Veronese N, Koyanagi A, Cereda E, et al. Sarcopenia reduces quality of life in the long-term: longitudinal analyses from the English longitudinal study of ageing. *Eur Geriatr Med.* 2022;13(3):633-639. doi: 610.1007/s41999-41022-00627-41993. Epub 42022 Feb 41925.
5. Li H, Li C, Wang A, et al. Associations between social and intellectual activities with cognitive trajectories in Chinese middle-aged and older adults: a nationally representative cohort study. *Alzheimers Res Ther.* 2020;12(1):115. doi: 110.1186/s13195-13020-00691-13196.
6. Igarashi T. Development of the Japanese version of the three-item loneliness scale. *BMC Psychol.* 2019;7(1):20. doi: 10.1186/s40359-40019-40285-40350.
7. Zhou P, Wang S, Yan Y, et al. Association between chronic diseases and depression in the middle-aged and older adult Chinese population-a seven-year follow-up study based on CHARLS. *Front Public Health.* 2023;11:1176669.(doi):10.3389/fpubh.2023.1176669. eCollection 1172023.
8. Obuchowski NA, Bullen JA. Receiver operating characteristic (ROC) curves: review of methods with applications in diagnostic medicine. *Phys Med Biol.* 2018;63(7):07TR01. doi: 10.1088/1361-6560/aab1084b1081.
9. Belkin M, Hsu D, Ma S, Mandal S. Reconciling modern machine-learning practice and the classical bias-variance trade-off. *Proc Natl Acad Sci U S A.* 2019;116(32):15849-15854. doi: 15810.11073/pnas.1903070116. Epub 1903072019 Jul 1903070124.
10. Vickers AJ, Elkin EB. Decision curve analysis: a novel method for evaluating prediction models. *Med Decis Making.* 2006;26(6):565-574. doi: 510.1177/0272989X06295361.
11. Li W, Guo Q. Plotting receiver operating characteristic and precision-recall curves from presence and background data. *Ecol Evol.* 2021;11(15):10192-10206. doi: 10110.11002/ece10193.17826. eCollection 12021 Aug.
12. Li J, Liu S, Hu Y, Zhu L, Mao Y, Liu J. Predicting Mortality in Intensive Care Unit Patients With Heart Failure Using an Interpretable Machine Learning Model: Retrospective Cohort Study. *J Med Internet Res.* 2022;24(8):e38082. doi: 38010.32196/38082.
