# Supplementary figures and images for: Machine Learning-Driven Personalized Risk Prediction: Developing an Explainable Sarcopenia Model for Older European Adults with Arthritis
Source: J Clin Med. 2026 Jan 27;15(3):1022. doi: 10.3390/jcm15031022 (PMC12897810; doi:10.3390/jcm15031022)

**Supplementary Table S2.** Random-forest imputation

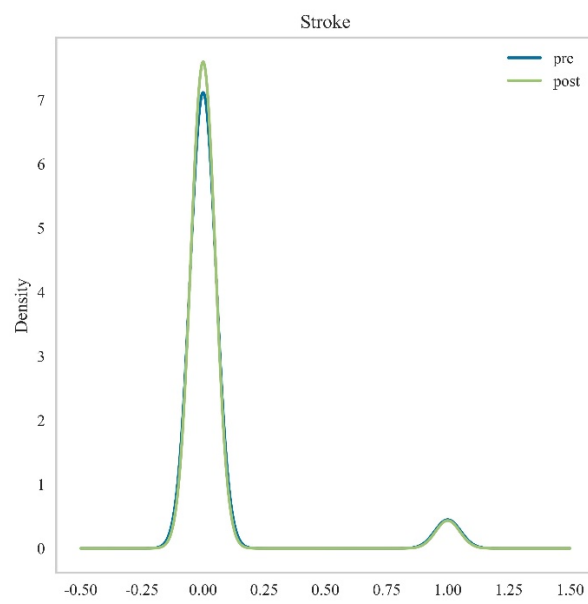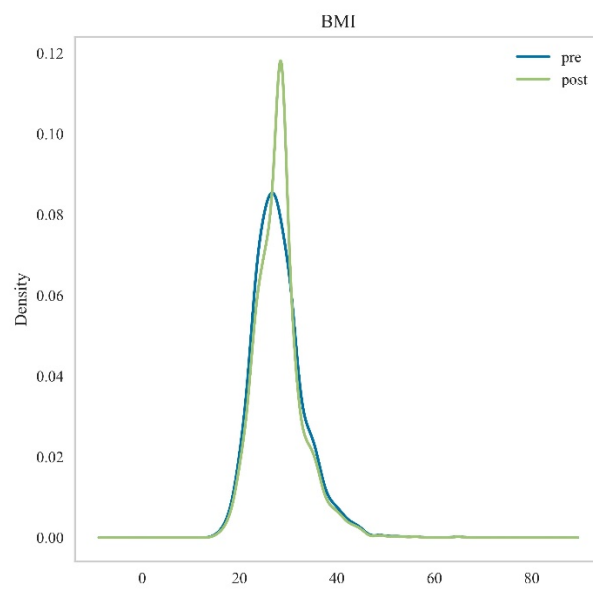

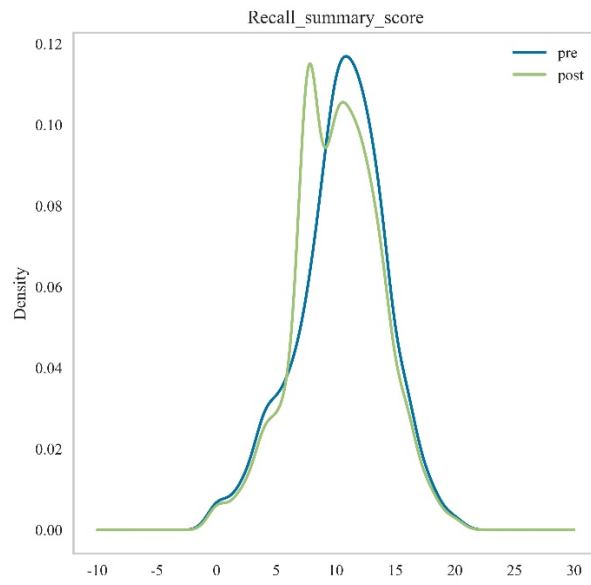

Supplement: Supplementary file 1 [file jcm-15-01022-s001.zip › Supplementary_tables/Supplementary_File_TableS2_RF_inputation.pdf]

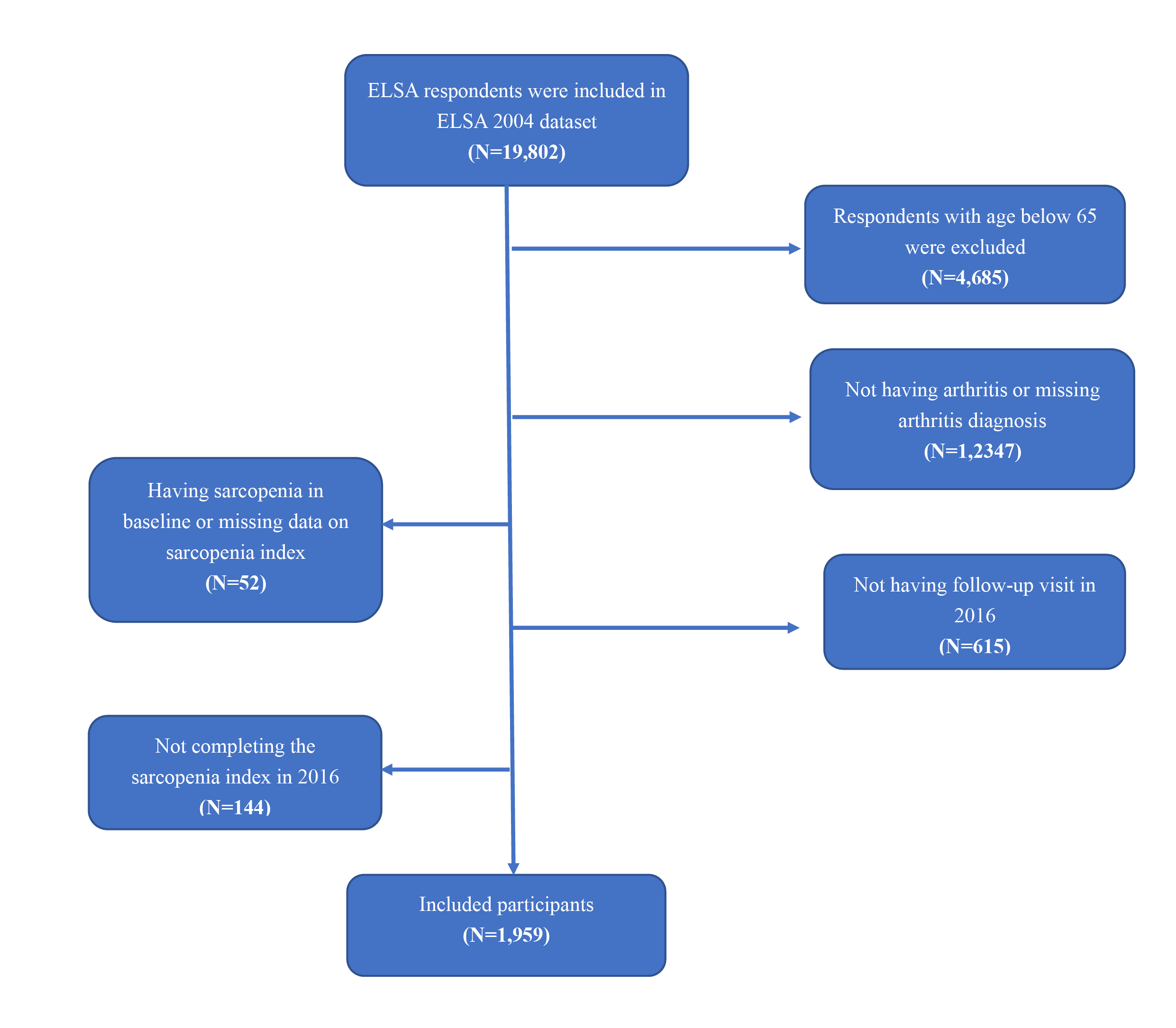

Supplement: Supplementary file 1 [file jcm-15-01022-s001.zip › Supplementary_figures_revise_OK/FigS1_ELSA.tif]

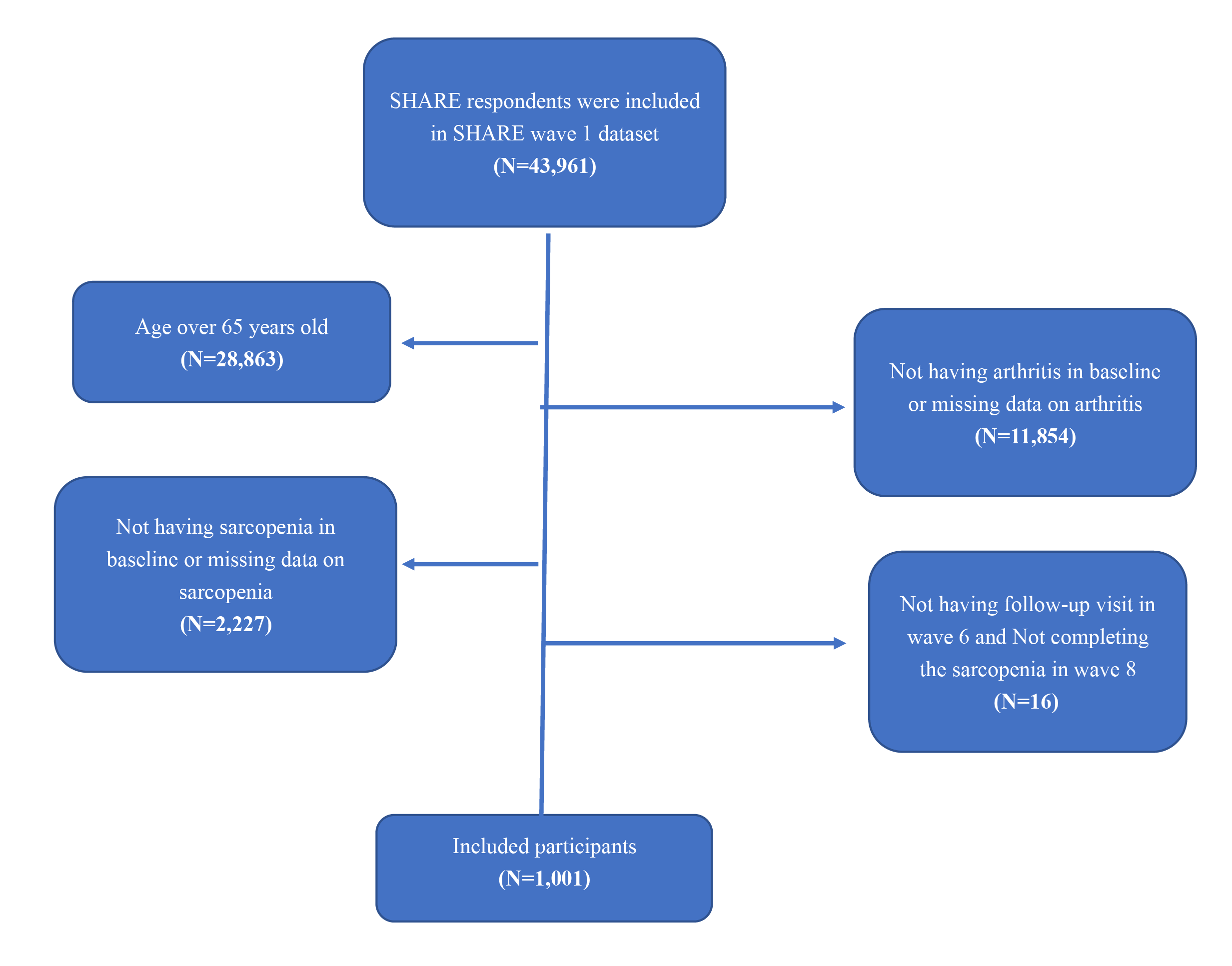

Supplement: Supplementary file 1 [file jcm-15-01022-s001.zip › Supplementary_figures_revise_OK/FigS2_SHARE_.tif]

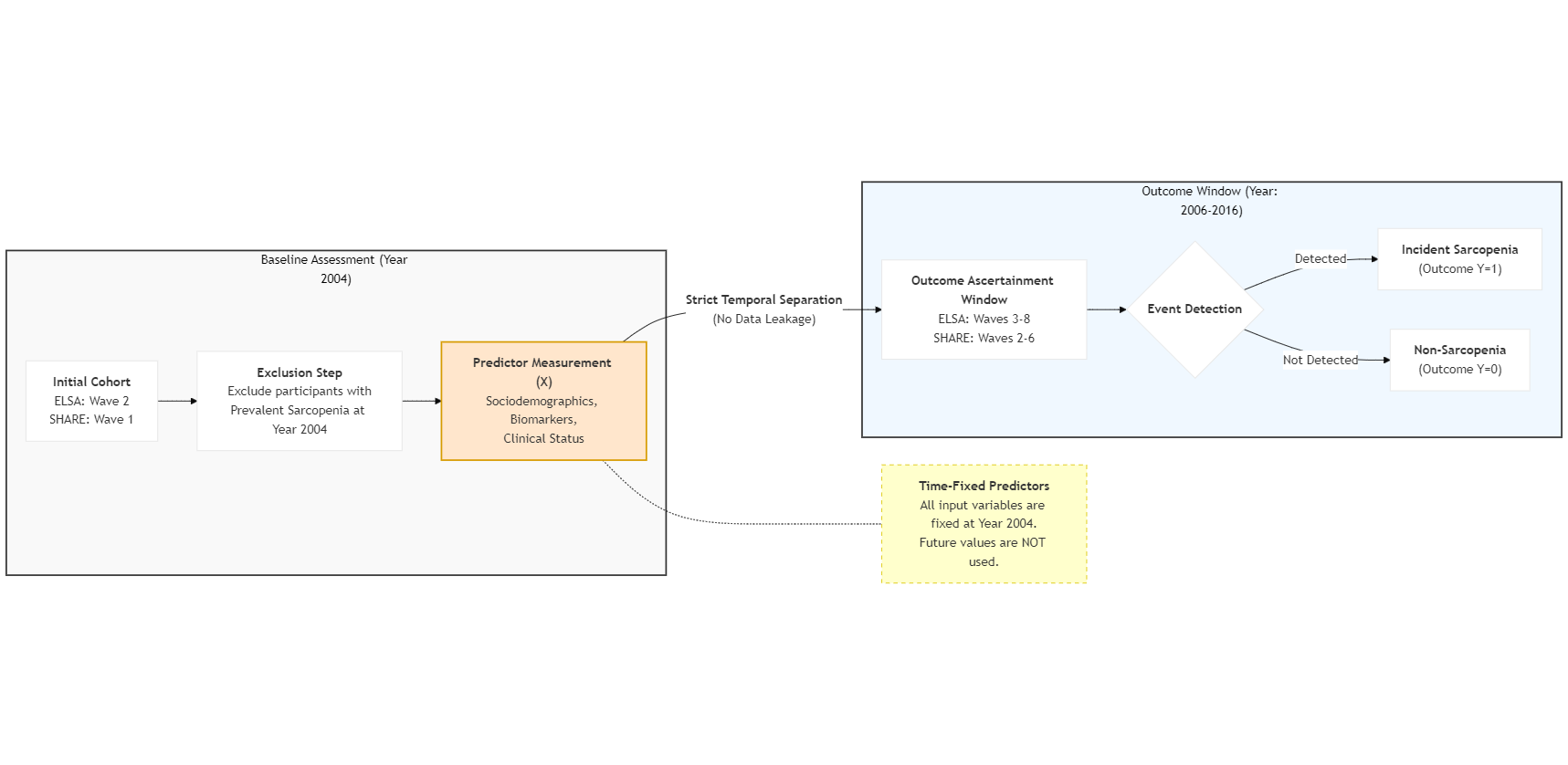

Supplement: Supplementary file 1 [file jcm-15-01022-s001.zip › Supplementary_figures_revise_OK/FigS3_prospective_longitudinal_study_design.png]

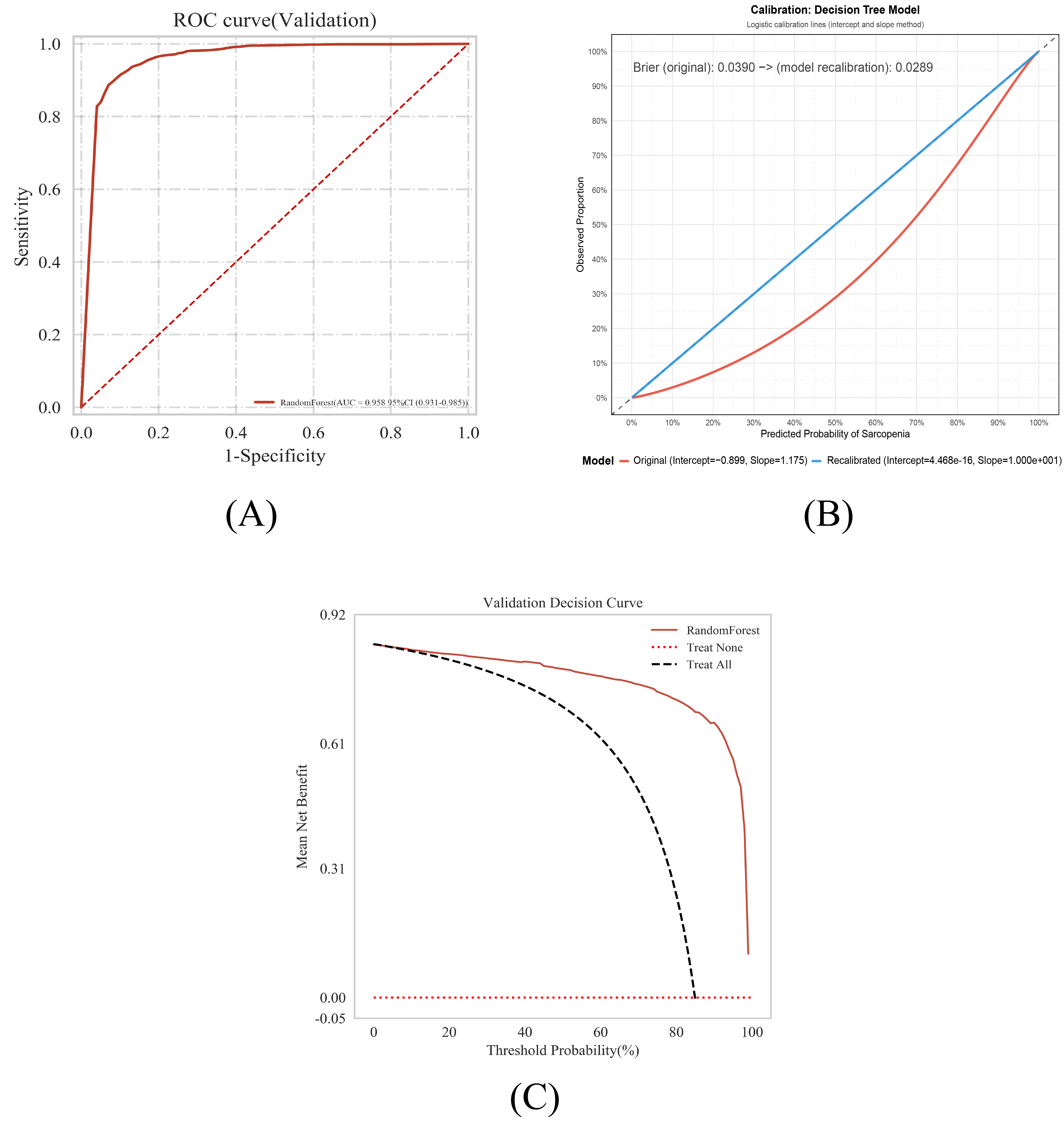

Supplement: Supplementary file 1 [file jcm-15-01022-s001.zip › Supplementary_figures_revise_OK/FigS4_Share_external.tif]
